# Identifying target areas for risk-based surveillance and control of transboundary animal diseases: a seasonal analysis of slaughter and live-trade cattle movements in Uganda

**DOI:** 10.1038/s41598-023-44518-4

**Published:** 2023-10-30

**Authors:** Lina González-Gordon, Thibaud Porphyre, Adrian Muwonge, Noelina Nantima, Rose Ademun, Sylvester Ochwo, Norbert Frank Mwiine, Lisa Boden, Dennis Muhanguzi, Barend Mark de C. Bronsvoort

**Affiliations:** 1 The Epidemiology, Economics and Risk Assessment (EERA) Group, The Roslin Institute at The Royal (Dick) School of Veterinary Studies, University of Edinburgh, Easter Bush, Midlothian, EH25 9RG UK; 2 Global Academy of Agriculture and Food Systems, Royal (Dick) School of Veterinary Studies and The Roslin Institute, University of Edinburgh, Easter Bush, Midlothian, EH25 9RG UK; 3 Laboratoire de Biométrie et Biologie Évolutive, UMR 5558, Universite Claude Bernard Lyon 1, CNRS, VetAgro Sup, Marcy l’Étoile, France; 4 The Digital One Health Laboratory, The Roslin Institute at The Royal (Dick) School of Veterinary Studies, University of Edinburgh, Easter Bush, Midlothian, EH25 9RG UK; 5 Department of Animal Health, Ministry of Agriculture Animal Industry and Fisheries, Entebbe, Uganda; 6 Center for Animal Health and Food Safety, College of Veterinary Medicine, University of Minnesota, Saint Paul, MN 55108 USA; 7 Department of Biomolecular Resources and Biolaboratory Sciences (BBS), College of Veterinary Medicine, Makerere University, Kampala, Uganda

**Keywords:** Risk factors, Infectious diseases

## Abstract

Animal movements are a major driver for the spread of Transboundary Animal Diseases (TADs). These movements link populations that would otherwise be isolated and hence create opportunities for susceptible and infected individuals to meet. We used social network analysis to describe the seasonal network structure of cattle movements in Uganda and unravel critical network features that identify districts or sub-regions for targeted risk-based surveillance and intervention. We constructed weighted, directed networks based on 2019 between-district cattle movements using official livestock mobility data; the purpose of the movement (‘slaughter’ vs. ‘live trade’) was used to subset the network and capture the risks more reliably. Our results show that cattle trade can result in local and long-distance disease spread in Uganda. Seasonal variability appears to impact the structure of the network, with high heterogeneity of node and edge activity identified throughout the seasons. These observations mean that the structure of the live trade network can be exploited to target influential district hubs within the cattle corridor and peripheral areas in the south and west, which would result in rapid network fragmentation, reducing the contact structure-related trade risks. Similar exploitable features were observed for the slaughter network, where cattle traffic serves mainly slaughter hubs close to urban centres along the cattle corridor. Critically, analyses that target the complex livestock supply value chain offer a unique framework for understanding and quantifying risks for TADs such as Foot-and-Mouth disease in a land-locked country like Uganda. These findings can be used to inform the development of risk-based surveillance strategies and decision making on resource allocation. For instance, vaccine deployment, biosecurity enforcement and capacity building for stakeholders at the local community and across animal health services with the potential to limit the socio-economic impact of outbreaks, or indeed reduce their frequency.

## Introduction

With an estimate of 15.5 million cattle heads, ~ 217,000 tonnes of beef and 2.8 billion litres of milk produced per year^1–3^, Uganda is a livestock-rich country with an increasing interest in strengthening its livestock systems^4^. Initiatives led by the government and supported by non-governmental institutions work on multiple fronts towards optimised and sustainable beef and dairy farming systems that enhance food security and boost agricultural economic development^4,5^. However, a high incidence of infectious diseases continuously threatens cattle production, with potential negative animal welfare, socio-economic, and public health implications^6^.

Understanding the factors that contribute to the spread of infectious diseases is a priority for an effective disease preparedness, prevention, and control. It is well-known that epidemics of animal infectious diseases are associated with animal movements, as these underlying networks connect susceptible and infected animals, and this can favour short and long-distance disease spread^7,8^. Transboundary animal diseases (TADs) are highly contagious, and transmissible infections linked to epidemics with the potential to spread rapidly to new areas, causing large-scale damage within and across countries^9^. Foot-and-mouth disease (FMD), Bluetongue, Contagious Bovine Pleuropneumonia (CBPP), Lumpy Skin Disease (LSD), and Rift Valley Fever (RVF) are examples of TADs listed by the World Organization of Animal Health (WOAH, founded as OIE) with evidence of circulation in Ugandan livestock^6,10–18^. Ongoing control programmes exist for FMD and CBPP, although their progress is limited to date, partially due to the complex socio-epidemiological situation influencing disease dynamics at the local level and budget restrictions that hinder widespread implementation of disease prevention and control measures^19^.

The role of cattle movements on TADs spread has been widely studied. In Africa, recent research highlights unique features and drivers of livestock mobility within constantly developing and diverse livestock systems^20–24^. Grazing mobility (e.g., pastoralism or nomadism) and trade-related mobility (e.g., farm-to-farm, farm-to-market, market-to-market, market-to-slaughterhouse, farm-to-slaughterhouse) are the two components of the livestock movement network in Uganda. While grazing mobility is a huge component of the cattle movement network, studying it requires the use of participatory approaches (e.g., surveys, focus groups) or cattle tracking^25^. In contrast, country-wide analyses of trade-related mobility can rely on the information extracted from official movement permits for a general overview of the system. The latter has become a popular approach to study animal networks that coincides with improved identification and traceability systems for livestock implemented in some African countries^24,26^.

Under the Ugandan Animal Disease Act 1963 (Amended in 2001), all livestock movements occurring between sub-counties or districts, or with an international partner, should be authorised by trained veterinary staff or the Commissioner Animal Health to ensure that requirements for the movements are met (e.g., ownership and health assessment) and to issue a short-term Animal Movement Permit (AMP) for an specific route and destination^27^. AMPs are implemented to improve the traceability of animal transactions, support disease control programmes, and reduce livestock theft. However, while current regulations do not include husbandry-linked movements for local livestock grazing or watering purposes, this data still provides the opportunity for a better understanding of cattle mobility in Uganda. It allows to explore the relationship with naturally occurring livestock production zones and possible risk pathways for the spread of TADs, taking into account seasonal variations and complex market dynamics.

In Africa, there is evidence showing that cultural practices, husbandry systems, herd size, distribution of food and water resources, cattle prices, and market activity influence the timing and volume of live cattle trade and grazing movements, and with it the risk of disease spread^21,28^. In contrast, because slaughterhouses often constitute sinks in cattle networks, little attention has been paid to slaughter movements, its seasonal calendar, and the possible role of key slaughter zones as active risk pathways for TADs transmission. A few studies document possible risk pathways for TADs in slaughter livestock, discussing the role of subjacent slaughter networks and their unique features linked to alternative livestock production systems in East Africa^29,30^. Thus, unravelling the architecture, cohesiveness, and district centrality network based on country-wide cattle movement traffic offers an opportunity for intelligent monitoring of TADs and can contribute to an integral outbreak response^31,32^. It not only provides the foundation on which tools such as pathogen genomic epidemiology and serologic landscaping of infectious diseases can be anchored, but it is also a framework for risk-based surveillance and data-driven decision making for managing outbreaks of TADs across the country. This is of great interest for smart allocation of resources towards a feasible, sustainable, and cost-effective disease prevention and control.

The aim of this study was to comprehensively characterise the network structure of cattle movements in Uganda based on official data collected in 2019. We considered this data to be an unaltered, representative overview of cattle movements before COVID-19 disrupted routine trading operations, distorting the collection of mobility data to date^33,34^. First, the annual static network is analysed as a snapshot of the cattle movements. We then describe the seasonal differences occurring between the dry and the wet periods for the live trade and slaughter networks due to hypothesised changes in cattle movement patterns. Lastly, we propose a centrality-based classification system to identify key areas for targeted disease surveillance and for the implementation of prevention and control interventions.

## Methods

### Study area

**Figure 1:**
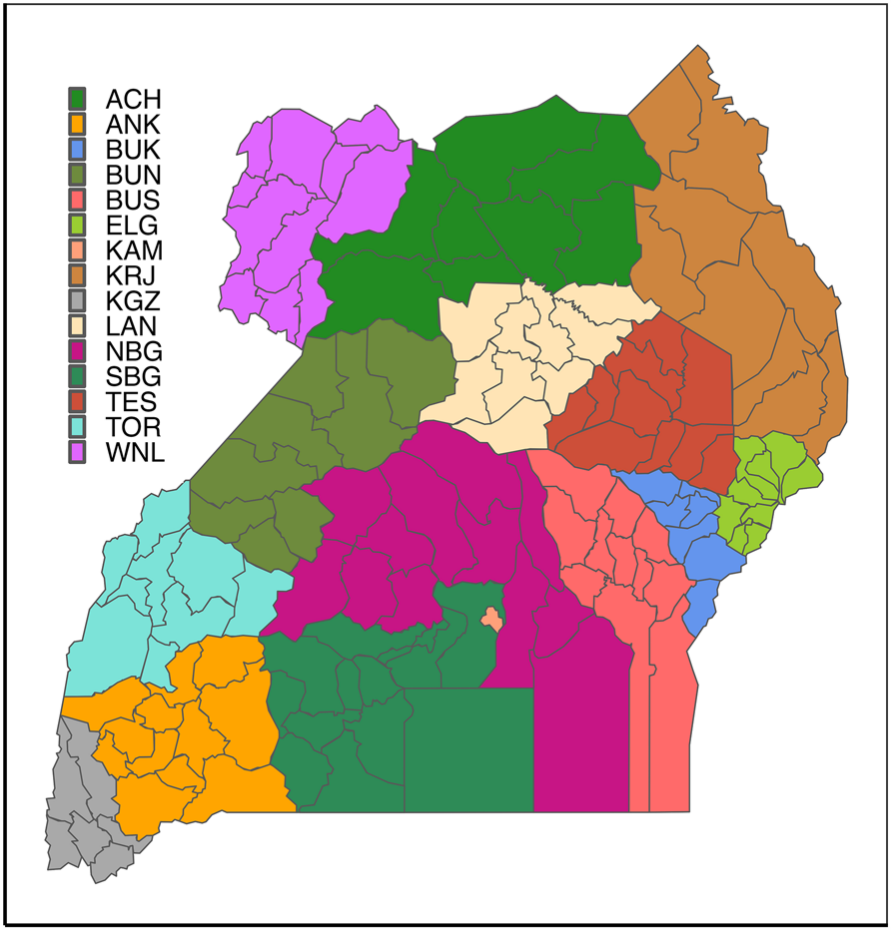
Sub-regions in Uganda. ACH, Acholi; ANK, Ankole; BUK, Bukedi; BUN, Bunyoro; BUS, Busoga; ELG, Elgon; KAM, Kampala; KRJ, Karamoja; KGZ, Kigezi; LAN, Lango; NBG, North Buganda; SBG, South Buganda; TES, Teso; TOR, Toro; WNL, West Nile.

Uganda is a land-locked East African country with a geographical extension of approximately 241,500 km^2^; it shares borders with Kenya, South Sudan, Tanzania, Rwanda and Democratic Republic of the Congo^35^. The country is divided into four major regions (Northern, Central, Eastern and Western) and 15 sub-regions (Acholi, Ankole, Bukedi, Bunyoro, Busoga, Elgon, Kampala, Karamoja, Kigezi, Lango, North and South Buganda, Teso, Toro and West Nile) (Fig. 1). Districts are the highest administrative units to date; 135 districts are officially recognised^36^. In general terms, Uganda has a tropical climate. The mean annual temperature and precipitation are, respectively, 22.4 °C and 1200 mm, with two dry and two rainy seasons per year^37^. The dry seasons are roughly from December to February and June to August, whereas the wet months, characterised by a continuous rainy pattern, usually occur between March and May (short rains) and from September to November (long rains)^37^.

### Cattle movement data

Cattle movement data officially sourced from the Ministry of Agriculture, Animal Industry and Fisheries (MAAIF) in Uganda was analysed; the records comprised movements that occurred within and in-between districts, covering a 1-year period between January 1st, 2019, and December 31st, 2019. The registry, based on animal movement permits (AMP) issued by the District Veterinary Officer (DVO) or an authorised delegate, included the date, origin and destination, type of transport, purpose of the movement, and the number of animals involved in the transfer. The date included on the AMP is the date of issue of the permit. In most cases, this corresponds exactly with the date of the movement, although for permits issued to farmers or cattle traders to move animals to livestock markets, the AMP may be issued a day or two before animals are sent to the markets and expires on the market day. Age and sex were also available, although with a high number of missing or invalid entries. Accuracy checks were performed in the data, including the verification of district nomenclature to map all the entries to the official administrative structure for 2019. All between-district AMPs in the original database were kept for analysis (n = 22,024).

### Network construction

Static, directed networks based on between-district movements for the purpose of live trade or slaughter were separately constructed for the whole study period (2019). Districts with no official report of between-districts movements were assumed to be inactive in the networks (Supplementary Information: Figure S1). The district of origin and destination of the movement were the nodes, and the movements between these were the edges (trade links). The sum of the number of animals moved from district-to-district (trade volume) was used as the weight of the directed edge. In addition, due to hypothesised temporal variability of cattle movements, static, directed, and weighted ‘dry period’ and ‘wet period’ networks were constructed to represent major seasonal changes occurring throughout the year. Seasonal changes are thought to impact trading patterns, and hence, these static networks are designed to capture potential variability in network architecture and cohesiveness^38–40^.

### Data analysis

#### Description of the Ugandan cattle movement network structure

The topology and connectedness of the annual and seasonal networks were described according to the parameters defined in the Supplementary Information: Table S1. The number of nodes and edges and, the density, reciprocity, and degree assortativity were used to document the general structure of the network, emphasising the differences between the annual and seasonal snapshots. Similarly, the average path length and the network diameter were the metrics used to capture the overall distance between districts^7^. These, in conjunction with the global transitivity coefficient, the identification of giant components, and the detection of within-network communities, were employed to describe how connected and clustered the entire cattle movement system in Uganda is. The Walktrap algorithm, based on the principle of random walks^41,42^, was used to identify the communities. In this study, communities refer to sub-groups of districts in which the concentration of edges within groups outnumbers the concentration of edges found between groups^42^; the community structure goodness-of-fit was assessed through the computation of the modularity score for each proposed community partition. The degree distribution and the relationship between the clustering coefficient score and the average shortest path length were examined; these concepts are useful to characterise if the network structure impacts disease spread. Formally, these are known as the ‘scale-free’ and ‘small-world’ properties of the network. Here, these concepts were assessed according to the standards proposed by Clauset et al.^43^ and Humphries & Gurney^44^, respectively.

#### Seasonality of Uganda’s cattle movement network

The extent to which the annual network accurately represents the structure of the system across the seasons was assessed through the comparison of the number of paths between these networks. This idea is expressed by the ‘Causal fidelity’ concept (*c*), a ratio measure quantifying the error of the static representation of a temporal network. Lower *c* values (*c* ≈ 0) indicate that the static network is a poor representation of the accessibility between pairs of nodes in the temporal network, which can be interpreted as the system not being static^45^. Based on this, we calculated the causal error (ε = 1/*c*) for the live trade network as a measure to estimate the worst-case outbreak scenario over the whole year. Moreover, the tendency of nodes and edges to remain active across the seasons was explored. Node loyalty (φ) and edge loyalty (θ) range from 0 to 1, in which φ or θ = 1 suggest an exact match (total preservation) of nodes or edges between the seasonal networks compared^45,46^.

#### Identification of influential districts on the Ugandan cattle movement network

The role of individual districts within the network was characterised using different global and local centrality measures. In-degree, out-degree, in-strength, out-strength, betweenness and PageRank centrality were used; these measures reflected the capacity of a district to be ‘influential’ when analysed in relation to the topology of its direct and indirect connections^47^.

Degree centrality describes the number of contacts per district. Central districts are those connected to many others. Out-degree refers exclusively to outgoing contacts, whereas in-degree relates to incoming contacts. Likewise, the strength of a node represents the sum of weights of all edges connected to a particular district, giving an indication of the volume of trade. In and out-strength can be interpreted accordingly. Betweenness centrality describes the frequency in which a district lies in the shortest path between pairs of districts across the network. In practical terms, districts with high betweenness act as bridges within the network. PageRank centrality assigns a weighed score representing both the number of direct connections per node and the degree of these connections; hence, due to its conceptualisation, it detects influential districts across the whole network (global metric). Weighted versions of betweenness and PageRank centrality were calculated; the normalised betweenness scores were reported. Spearman’s ρ was used to produce a correlation matrix between the centrality measures assessed. In addition, seasonal differences in centrality scores were evaluated by conducting hypothesis tests through a permutation approach. Permutation tests have been successfully used to test independence hypothesis and to verify causal attributions in network analysis^21,48^.

A classification system for districts according to their centrality profile was devised for the live trade network; this analysis was conducted through Hierarchical Clustering on Principal Components (HCPC). Local transitivity scores, calculated for each district, were included as one of the input variables to account for the network tendency to cluster. Principal Component Analysis (PCA) was performed as a reduction technique to identify uncorrelated components describing the variation in the data^49,50^. Thereafter, Hierarchical Cluster Analysis (HCA) was conducted; altogether this approach looked to detect groups of districts that share similar characteristics based on the combination of ranked centrality scores. The identification of ‘prominent’ districts following a multivariable approach is valuable from an epidemiological perspective to identify key districts for infectious disease surveillance and control purposes.

#### Network resilience to identify districts for intervention

The impact of targeted district removal on the vulnerability of the seasonal networks was tested through sequential deletion of districts from the trading system according to their ranked centrality scores. In this study, the efficacy of district removal was quantified through the computation of the largest connected components (GCCs). The ‘Giant Strongly Connected Components’—GSCC and the ‘Giant Weakly Connected Components’—GWCC reflect the connectedness of the network and, from an epidemiological perspective are important proxy indicators of the epidemic size for a newly introduced infectious agent^51^; in this case, a recently identified disease outbreak. Targeted centrality-based district removal was compared to at-random district deletion and to a ‘gold standard’ district prioritisation scheme based on the greedy algorithm as proposed by Ciccolini et al.^52^. Briefly, the greedy percolation algorithm optimises the order in which districts are selected for removal such that the addition of sequential districts minimises the size of the GCC. In practice, these strategies represent the implementation of targeted strategies to tackle disease spread. Widespread pre-emptive or post-outbreak vaccination and reinforcement of surveillance systems are examples of possible approaches employed before or after the identification of disease outbreaks at the district level.

All the analyses were conducted in the R environment using RStudio^53^; packages used included *igraph, qgraph and ggplot2*.

## Results

### Ugandan cattle movements

**Figure 2:**
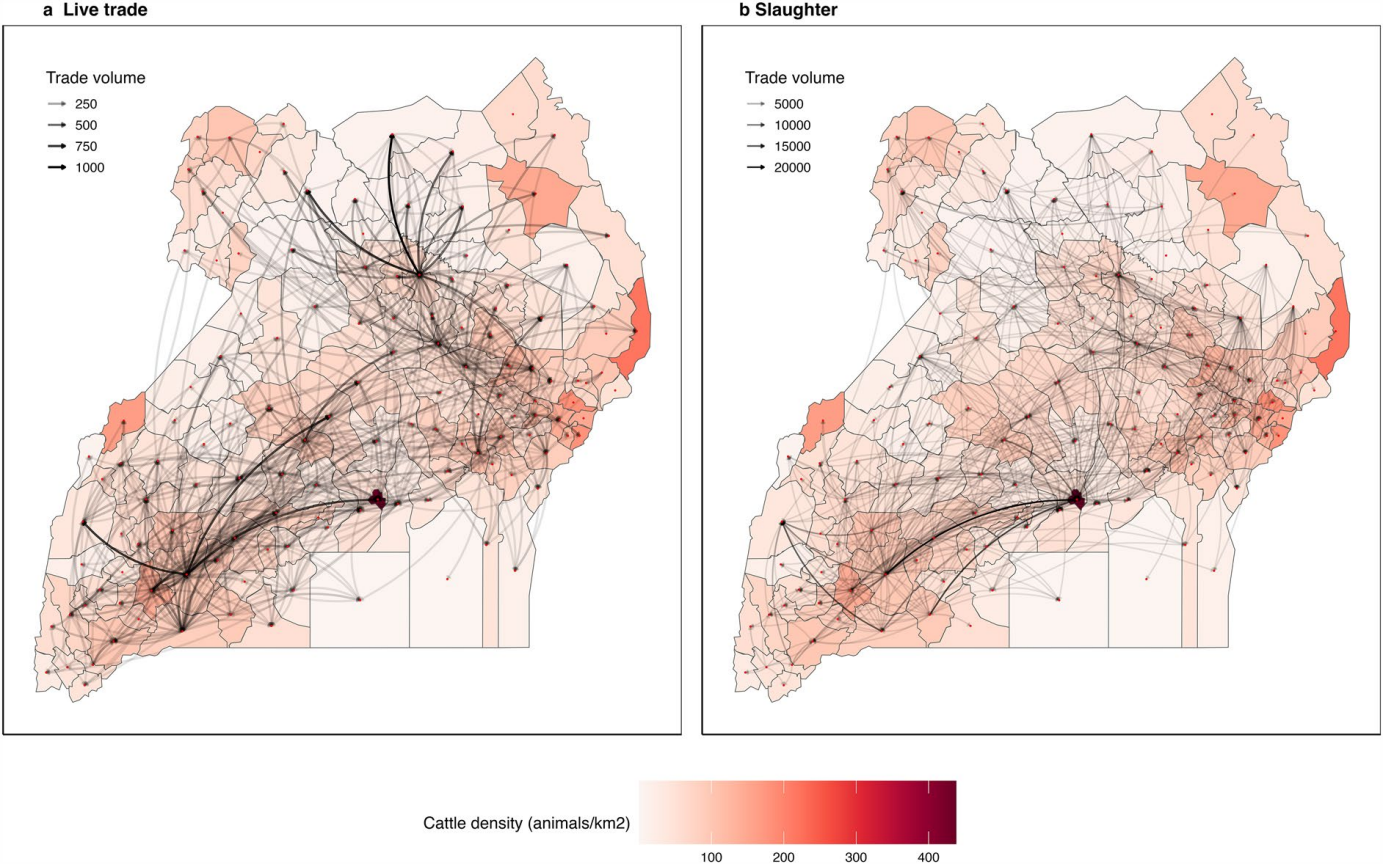
Cattle movement networks in Uganda, 2019. Between-district movements are weighted based on trade volume. (**a**) Live trade and (**b**) Slaughter.

**Figure 3:**
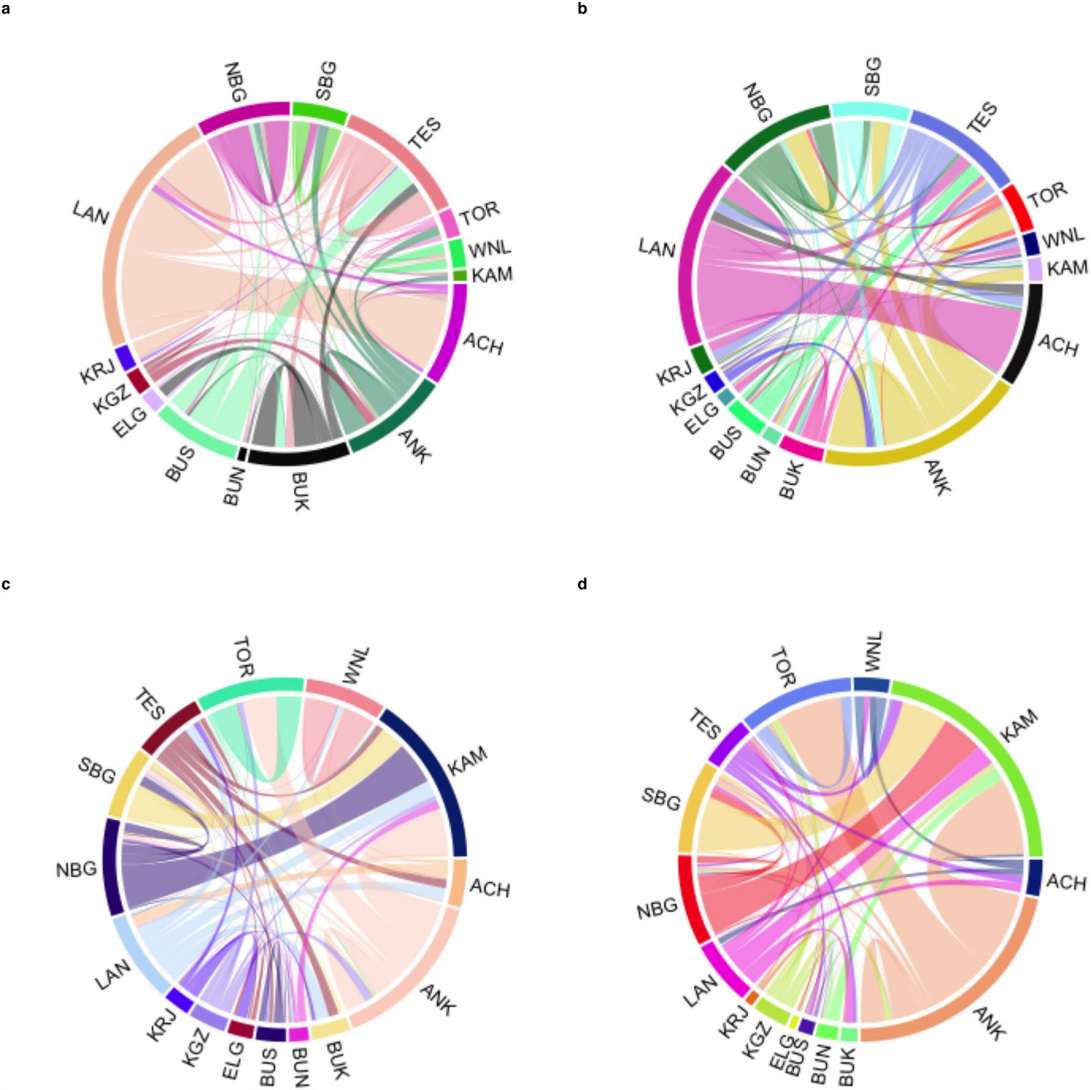
Cattle mobility across the 15 sub-regions for the slaughter and live trade cattle networks in 2019. Interconnections between sub-regions can be traced by the coloured line segment (chord) whose endpoints lie in the sub-region of origin and destination of the cattle movement. The contrast between the number of batches moved and the volume of trade is depicted in the chord diagrams: (**a**) Live trade batch movement permits, (**b**) Live trade volume, (**c**) Slaughter batch movement permits and (**d**) Slaughter volume. ACH, Acholi; ANK, Ankole; BUK, Bukedi; BUN, Bunyoro; BUS, Busoga; ELG, Elgon; KAM, Kampala; KRJ, Karamoja; KGZ, Kigezi; LAN, Lango; NBG, North Buganda; SBG, South Buganda; TES, Teso; TOR, Toro; WNL, West Nile.

For the period of 2019, 5114 and 16,910 between-district batch movements were officially registered for live trade and slaughter (Fig. 2). These movements involved a total of 234,229 animals moved mainly for slaughter purposes (87.65%). While trucks were the predominant vehicle used to transport cattle to livestock markets, from farm-to-farm and to slaughterhouses (79.93%), it is noteworthy that a small proportion of cattle were moved on boats (0.25%) and smaller vehicles (19.82%) (e.g., cars, motorcycles, and bicycles). A comparative description of the batch sizes and number of animals transported by sex, age, purpose and means of transportation is shown in the Supplementary Information: Table S2. Movements edges indicated that cattle mobility in Uganda was highly asymmetrical; most cattle transactions tended to remain local with a clear geographical pattern showing that the movements frequently occurred within the sub-regions. There were disproportionately more live trade movements within or from districts in the Teso, Lango and Ankole sub-regions as shown in Fig. 3. Kampala was the largest sink for slaughtered animals most of which originated from the central (Buganda), southwest (Ankole), and northern (Lango) parts of Uganda.

### Dynamics of cattle movements

**Figure 4:**
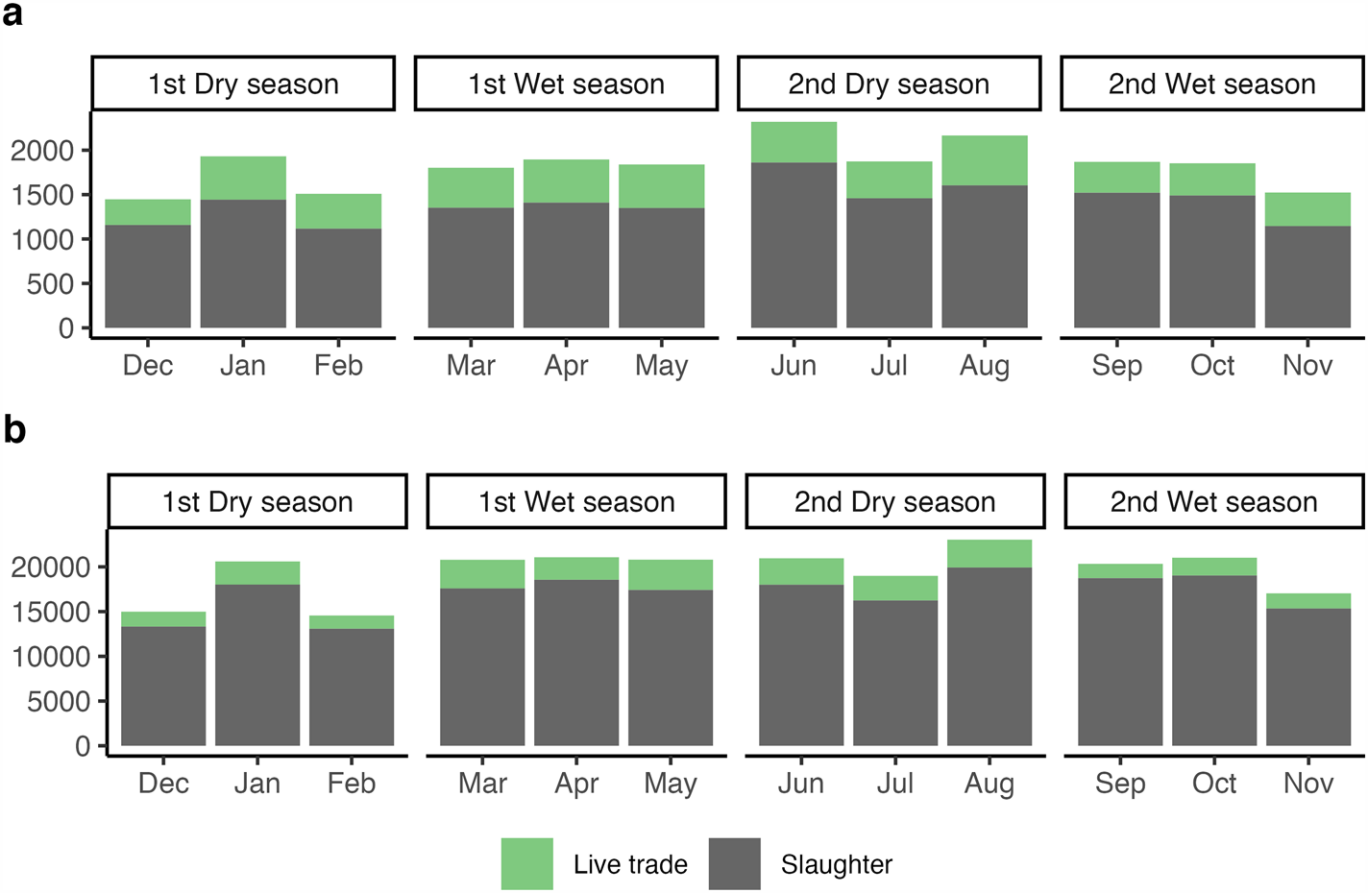
Monthly time series for cattle movements. The months comprising the dry and wet seasons are shown. (**a**) Number of batches and (**b**) Volume of trade.

Live trade movements peaked between March and August with ~ 1400 batches and over 8000 cattle moved; this corresponded to the 1st wet (March–May) and 2nd dry seasons (June–August). In contrast, slaughter-related movements remained stable across the seasons except during the 1st dry season (December to February) indicating decreased movement of animals for slaughter (Fig. 4). We identified variations in the districts that were active in the cattle network across the seasons as shown by the analysis of node similarity (node loyalty) (Supplementary Information: Figure S2), with more stability in the destination than the origin of cattle movements. For instance, node loyalty of the origin districts ranged between ~ 50% and ~ 70% across seasons (Live trade: 51–78%; Slaughter: 48–70%), whereas this value was over 70% for the destination in all periods (Live trade: 73–81%; Slaughter: 78%–84%). However, the structure of these connections varied across seasons. For live and slaughter trade, weighted and unweighted edge loyalty was below 10% for all seasons, indicating that the movement links between the districts and the volume of trade were variable. The comparison of the number of paths between static annual and seasonal networks revealed that the annual network is not a reliable representation of the seasonal system. For the live trade network, 42% of the paths that appear in the annual cattle movement network are not present in the seasonal networks. This value increases to 49% for the slaughter networks. In practice, this means that the annual live cattle trade network could overestimate the size of an outbreak by 1.72 (causal error) based on its actual seasonal accessibility.

### Network topology

Our results show that all architectural features of the seasonal cattle networks had lower values than for the annual network. Overall, a low connectivity characterised the annual live trade and slaughter networks (~ 4%) with a network density close to 2% for each period. The density of the live trade network was found to be at is lowest during the second wet season. Reciprocal links occurred more often in the live trade than in the slaughter network (17.4% vs. 12.0%) with a range between 8.8% and 10.8% in the live trade and 4.3% and 6.2% in the slaughter seasonal networks. In general, large degree-districts tended to preferentially attach to low-degree districts, as indicated by a negative degree assortativity value (disassortative networks). Details of the network topology for the live trade and slaughter networks are shown in the Supplementary Information: Tables S3 and S4.

### Cattle network cohesiveness

To describe the networks in terms of its cohesion, several measures were used to assess the level of district connectedness. This allowed us to highlight isolated districts, identify active trading communities, and explore the potential extension of TADs spread through cattle movements. Here, the networks displayed a low level of clustering, evidenced by transitivity coefficients close to 0.20 for the annual network as well as across the seasons. Lower transitivity scores during the second wet season suggest that during an epidemic, a disease would spread more slowly during this period when compared to the other seasons. However, both live trade and slaughter annual and seasonal networks exhibited small-world properties with transitivity scores substantially higher than the ones computed for random networks and average shortest path lengths similar or higher than the ones calculated for random networks of equivalent structure (Small-world Index ≥ 3) (See Supplementary Information: Table S5). Between 7 and 13 steps were required to connect the most distant districts in the live trade seasonal networks, while 8 steps were needed to get all the way across the annual network. In contrast, this value was reduced to 2 in all slaughter networks. On average, 2.69 and 2.91 steps were required to get from one district to another for the live and slaughter networks, respectively. The average path length was slightly larger in the seasonal networks (2.86–3.78 for live trade and 2.64–5.14 for slaughter).

As indicated by the size of the largest weakly connected component (GWCC), all districts reporting cattle movements in the studied period were involved in trade, forming one annual network through undirected paths. However, when directionality was considered, the global connectivity of the network was reduced, with about one third of the total districts directly connected for live animal trade (38.4%) and slaughter (30%). The larger structures formed by connected districts were further reduced in the seasonal networks; the number of undirected and directed components were heterogenous across the seasons, implying a high level of fragmentation. Across the seasons, networks were structured forming one or two weakly connected components. In contrast, several strongly connected components were identified per season, but also many districts were solitary, creating islands in the network. The largest size of the GSCC for the seasons was 28, 22, 9 and 8 for the first dry and wet and the second dry and wet periods respectively, which can be interpreted as a higher network tendency towards fragmentation during the second half of the year. Seasonally, the slaughter network was more fragmented during the wet season (GSCC 9 and 13 for wet seasons 1 and 2) than during the dry season (24 and 17 for the dry seasons 1 and 2).

**Figure 5:**
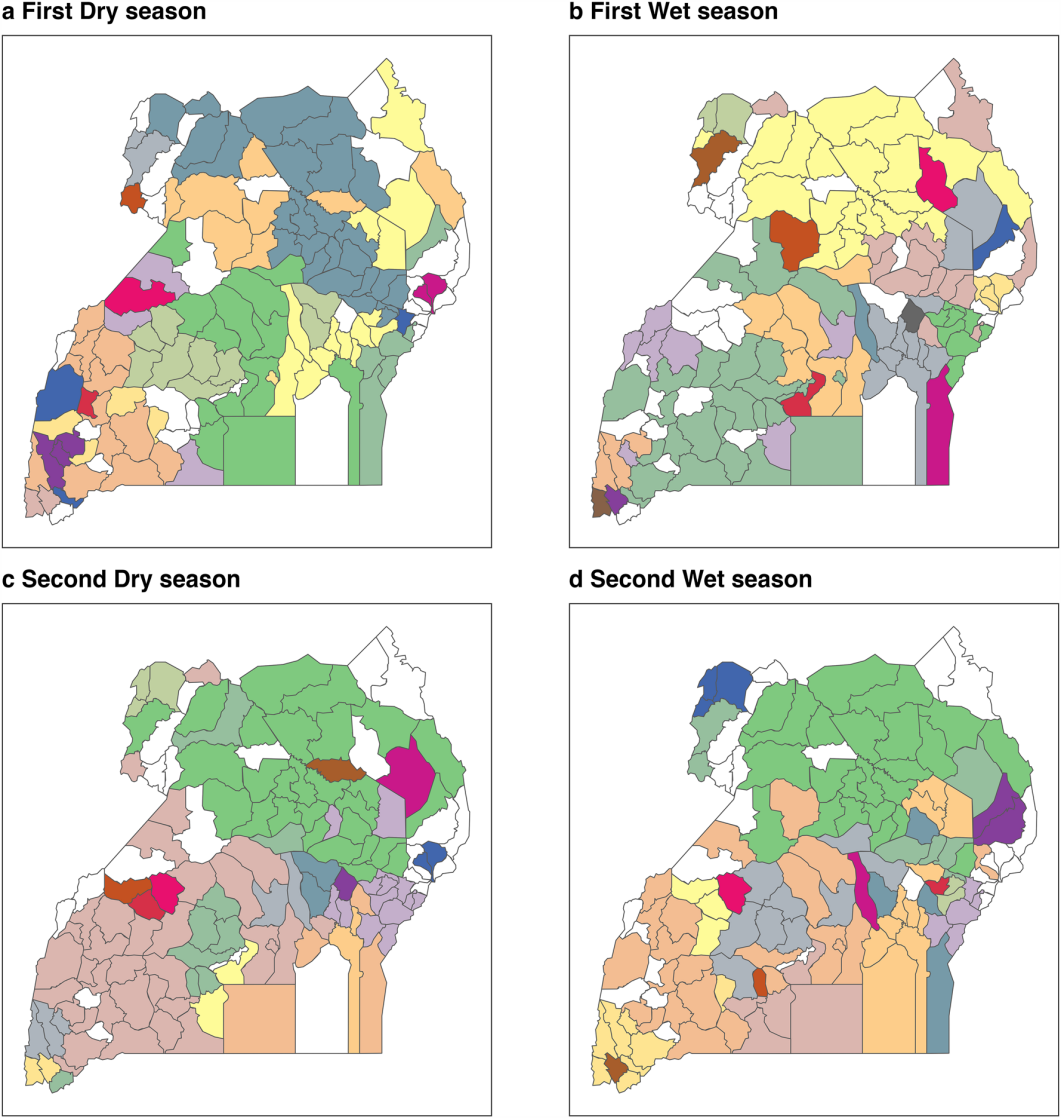
Live-trade network communities and its spatial distribution across the seasons. Districts in white were not active in the network for the assessed period. Each community is depicted by a unique colour.

The community structure algorithm applied to the annual movements revealed that the districts were organized in 11 (live trade) and 22 (slaughter) groups. Detection of communities across seasons was diverse, varying in number and distribution throughout the country; however, the groups of districts tended to be geographically clustered. For live cattle trade, modularity values suggested a moderate to strong community structure across seasons, with scores between 0.52 and 0.57 supporting the proposed network division (See Fig. 5). The largest and most stable community was in northern Uganda, covering the Acholi, Lango, Teso and Karamoja sub-regions. Likewise, a large community extended across the southwestern and central regions in all seasons. Smaller communities were detected towards the southeast, west and northwest of the country. In contrast, seasonal slaughter networks displayed lower modularity, except for the first dry season (December to February) (Supplementary Information: Table S4 and Figure S3).

### Influential districts

To estimate the importance and role of each district within the network, we used several measures to calculate district centrality and thereby their potential importance in relation to TADs flow in the whole system. The analysis of the network distribution shows that all networks (annual and seasonal) exhibited scale-free properties (Power-law distribution), with most districts showing very few connections and few districts acting as network hubs characterised by a larger number of links. Node centrality measures considered epidemiologically relevant for the live trade network were in-degree, out-degree, in-strength, out-strength, betweenness, and PageRank. In contrast, for the slaughter network, only in-degree, out-degree, in-strength, and out-strength were calculated to account for a small probability of local pathogen acquisition and onward transmission under diverse cattle slaughter schemes.

For live trade, a district was connected, on average, to 10.99 other districts (median 7; range 1–64), with a median of 5 incoming (range 0–25) and 0 outgoing connections (range 0–48) for the whole year. When accounting for the volume of trade (strength), the median number of cattle moved between districts was 217 (range: 1–6963), whereas cattle moved to and from districts ranged from 0–1318 and 0–6232, respectively. While district median degree was relatively steady across the seasons, some fluctuations in the volume of trade and the number of connections between districts were identified (See Supplementary Information: Figure S4), particularly for incoming links as indicated by the seasonal variation across the in-degree and in-strength values (*p* < 0.01). A higher average strength was identified between March and May (median 74; range 1–3143) and June to August (median: 67; range: 2–3087), the periods corresponding respectively to the first wet and second dry season. A slightly lower number of average incoming links to other districts was observed for the second wet season in comparison to other seasons (*p* < 0.01) (Supplementary Information: Table S6).

The contact structure was homogenous across districts in the slaughter network with similar average direct connections (degree, in-degree, and out-degree) across the seasons (*p* > 0.05) (Supplementary Information: Figure S4). On average, a district was linked to 11.02 districts across the country in the annual network (median: 8; range 1–68) with up to 42 and 61 inbound and outbound links to other districts, respectively. Moreover, the volume of trade of these links, as shown by the strength, was also uniform with no statistically significant differences identified across seasons (*p* > 0.05). In 2019, the average strength for all districts was 3158 (median: 528; range 1–93,623) (Supplementary Information: Table S6).

The geographical distribution of the top-ranked districts for each centrality measure for the live trade and slaughter networks is shown in the Supplementary Information: Figure S5 and S6. Although variation was identified, some districts were consistently ranked on top positions using all centrality metrics and kept their prominent position across the seasons. For slaughter, districts located across the cattle corridor were commonly among the top 5% for out-degree and out-strength, whereas districts with high in-degree and in-strength tended to be peripherally located, close to the borders in the northwest, west and southeast, or in densely populated areas (e.g., Kampala, Wakiso). Similarly, the highest ranked districts for live trade movements (out-degree and out-strength) were in the cattle corridor, with several influential districts located in Ankole and Buganda. In terms of destination, while most movement traffic occurred within the cattle corridor (in-degree), a high volume of trade was consistently directed towards the northern region in both wet seasons and the second dry season. Mapped top districts for betweenness and PageRank centrality also show a similar pattern across the seasons (Supplementary Information: Figure S5).

### Tailoring interventions to districts

**Figure 6:**
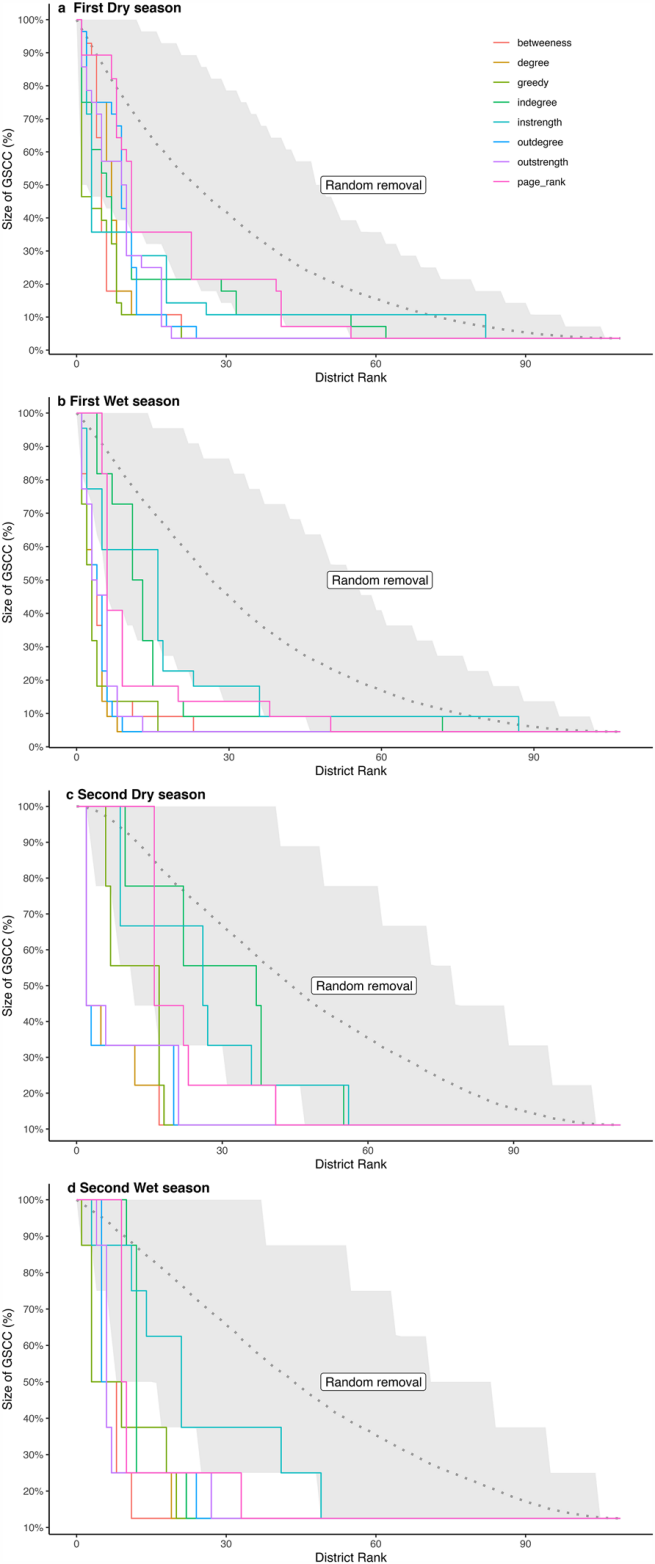
Plot of centrality-based percolation per season—Live trade. Performance measured through the proportional size of the giant strongly connected component (GSCC) after the removal of ranked districts.

Because centrality measures reflect the importance of each district within the cattle mobility network and can be considered a proxy for the district-level spreading capacity of TADs, we conducted a percolation analysis based on a centrality-targeting approach to identify the best strategy to preferentially allocate disease prevention and control interventions such that the probability of further pathogen spread is reduced. The percolation analysis was conducted independently per seasonal network. For the live trade network, district deletion per ranked in-degree, out-degree, in-strength, out-strength, betweenness and PageRank centralities was performed. For comparison, an algorithm randomly sorting out the deletion sequence of districts was created (1000 iterations). Results from the percolation analysis are interpreted in relation to the speed at which each centrality measure achieves network decomposition. In other words, how quickly is it possible to fragment the network, and as a result, decrease the maximum and minimum expected epidemic sizes (GWCC and GSCC, respectively). The seasonal networks were robust to random district removal. However, the deletion of districts ranked on their betweenness, out-strength and out-degree was consistently more efficient in terms of decreasing the GSCC and GWCC across the seasons (See Fig. 6 and the Supplementary Information for Figure S7).

### District centrality-based classification scheme based on live trade

**Figure 7:**
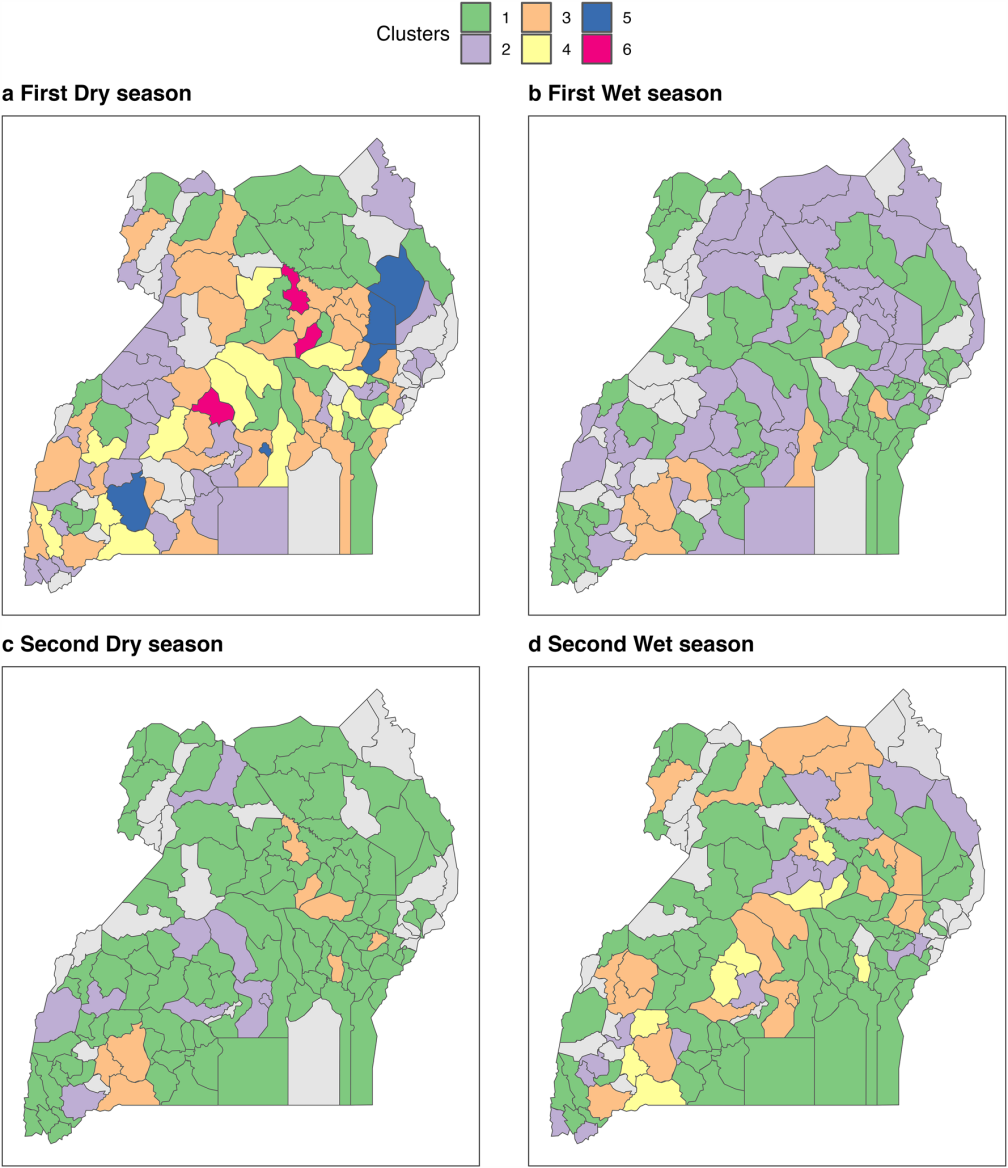
Allocation of districts to clusters based on centrality-based similarity profile per season—Live trade cattle network. Districts in light grey were not active in the network for the assessed period.

Hierarchical Clustering on Principal Components (HCPC) was performed on a selection of centrality scores. The analysis showed which districts have a similar importance profile per season. In-degree, out-degree, in-strength, out-strength, betweenness, and PageRank centralities were included. Local transitivity scores, computed per district, were added to the list of variables considered for the calculation of the composite classification system. In the dry seasons, districts were grouped into 6 and 3 classes for the first and second dry season, respectively. In contrast, during the wet season, 3 clusters for the first wet season and 4 for the second were identified. District identity according to the classification scheme is illustrated in Fig. 7.

While the cluster analysis produced different descriptions of the centrality-based relationships between the districts, it was possible to identify some common features. For instance, in the dry seasons, one of the clusters consistently represented districts with a tendency to high local cohesiveness (high local transitivity) associated with low centrality scores. In addition, another ‘common’ cluster represented the least connected, least important districts across the network, as indicated by its low local transitivity and low centrality values. Other clusters offered different combinations of centrality measures, providing an indication of their mixed role in the network. As indicated in the PCA, districts with high in-degree, in-strength and PageRank centrality were grouped together, representing districts extensively connected across the network with a large influx of cattle from several districts. In a similar way, districts with high out-degree, out-strength, and betweenness tended to be part of the same category; these districts connect parts of the network that would otherwise be disconnected while also dispatching large numbers of cattle to several locations. Lastly, some districts served a dual function in the network by displaying high betweenness and high PageRank centrality values.

In contrast, the two wet seasons were characterised by the identification of a cluster formed by the least influential districts in the network (low centrality and low local transitivity). Moreover, and like the classification found in the dry season, districts with high number of incoming connections, receiving a high volume of cattle, and with high PageRank values, were grouped together. The variables associated to each cluster and its description are presented in the Supplementary Information: Table S7.

## Discussion

Livestock movements are one of the major drivers of infectious disease spread as they shape the contact structure of animal populations, and with this, the transmission of pathogens. Hence, livestock movement permits are an important tool for veterinary services because they represent a critical unit of traceability for TAD prevention and control. By using information on livestock movement permits, it is possible to construct a movement network that approximates the country-wide cattle contact structure at a higher level. Network-based risk assessment can inform animal health policy by identifying key trading routes and high-risk areas in which more efficient onward disease transmission may occur and that can be targeted to reduce the burden of disease and the likelihood of pathogen spread. This study used network analysis to describe cattle mobility networks in Uganda. We hypothesised that the season (wet or dry) and reasons for moving animals (slaughter or live trade) impact the network structure and cohesiveness, and thus, the possibility of disease transmission and the effectiveness of outbreak prevention and response measures.

In Uganda, cattle-trading dynamics based on official cattle mobility data indicate that infectious diseases could spread widely across a temporally heterogenous movement network. Our results suggest that at the most conservative scenario, local and long-haul disease spread are likely to occur via live cattle trade, with up to 20% of the districts potentially becoming infected after an outbreak. Approximately three sequential between-district movements may connect susceptible and infected animals through live animal trade. This seems to dispel the notion that livestock may be protected by vast geographical distances. Indeed, long-distance transmission events of TADs such as FMD and Tsetse-transmitted human African trypanosomiasis (hAT) have been reported in Uganda^14,54–56^.

At a localised level, we note that disease transmission across spatially embedded, large-scale trading communities is possible (Fig. 5). Sizeable communities spanning the northern (mostly Acholi and Lango) and central regions (North and South Buganda) remained stable across the seasons, with smaller trading communities present in the southeast and southwest. From a surveillance point of view, this suggests that targeting the most influential districts in these communities has the potential to provide valuable information about the whole region. Similarly, prioritising disease control interventions in key districts at the local level could contribute to reducing the wider burden of TADs, hence unlocking the epidemic intelligence utility of cattle movement data in Uganda. If stable district communities were identified throughout the years, such an approach would support animal health regional planning^57^, constituting an integrated animal health structure in which record-keeping, surveillance, biosecurity and government inspection become key actions towards reducing the likelihood of disease spread across districts with strong commercial ties^58^.

At the country level, our results show that the cattle movement network was characterised by a high level of seasonal variability, likely representing a heterogenous country-wide accessibility. This is because seasonal networks were smaller, less connected, and characterised by lower values of most structural parameters evaluated (Supplementary Information: Table S3 and S4). This observation could indicate an underlying time dynamical system influencing cattle trade and slaughter, as has been described in similar settings^38^. The first dry and wet seasons were likely to be associated with larger epidemics, given the connectedness via the GSCC. We note that peak live trade volume occurred between March and May during the first wet season and from June to August in the second dry season, with decreasing cattle mobility and lower network density observed towards the end of the year. Seasonal variation in cattle supply chains has been documented in Uganda, and there are differences in the local livestock husbandry systems, agroclimatic factors, marketing chain structure and activity, price trends, herd structure dynamics and festivities which strongly influence cattle traffic trends across the country^40,55^. Livestock supply peaks with optimal prices and better-quality cattle becoming available for sale, and this boosts wide-ranging livestock mobility, resulting in a continuous and pulsating livestock trade environment with epidemiological implications for the country-wide dynamics of TADs and other infectious diseases. Therefore, increased mid-year cattle traffic coincides with an anticipated rise on sales of mixed type cattle for breeding, ploughing, or re-stocking amongst large-scale ranches and agro-pastoral farmers along the cattle corridor, the pastoral Karamojong and the smallholder agro-pastoral communities in peripheral districts along the west and southeast^39,40,56^. In contrast, the end-of-year decline in cattle movements could be associated to escalating livestock prices and an increased sale of aged animals to be culled during the festive season^40,55^.Therefore, the seasonality observed at national level partially explains the variations of the active districts, trade links and volumes.

Our results also show a high volume of livestock movements across the breadth of the country. In Uganda, livestock moved for sale, both within and outside of markets, follow a complex chain involving negotiations at the farm gate, primary, secondary and tertiary markets in a predominantly broker-mediated system that involves farmers, short and long-distance traders, as well as urban meat distributors^39,40^. High mobility and frequent animal mixing are a central feature of this dynamic livestock marketing system. Cattle traders often travel within and across districts in trucks for extended periods of time, simultaneously trading replacement stock for breeding, ploughing, fattening and animals for slaughter^39^. This creates the opportunity for disease transmission and makes districts constantly vulnerable to disease incursion.

While it is widely known that live animal trade is a major route for TADs spread in livestock, the role of slaughter-related trade has been poorly studied and often relegated to a lower priority when understanding disease patterns and transmission risks. In many countries, slaughterhouses are often considered epidemiological dead ends, supported by a reliable traceability system to maintain food safety standards and safeguard animal welfare. In contrast, cattle moved for slaughter in Uganda are marketed through a complex structure integrating several livestock production scales and systems within a multi-layered beef value chain. This system is supported by large abattoirs (mostly in Kampala), secondary urban slaughterhouses near major towns, town slaughter slabs and, on-the-ground and family slaughter at smaller local markets and households. It is possible that infected animals are slaughtered due to variable opportunities for disease detection, depending on the predominant livestock system and geographical accessibility. Infections can occur before (in farm), during (in transit) or after cattle sale (waiting time), boosted by frequent movements and lengthy waiting periods before slaughter^29,30^. Furthermore, onward disease transmission could occur in association to re-stocking from animals already sold for slaughter, a practice more likely to happen within local town markets, especially in agro-pastoral areas. These scenarios and the associated risk pathways across production systems in slaughter cattle have been recently described and modelled in East Africa^29,30^.

The differences in movement for slaughter or live animal sales are epidemiologically important but can be difficult to disentangle. Here, we observe that both live trade and slaughter seasonal networks were characterised by a dissimilar between-district contact structure (Fig. 3). Most districts displayed very few between-district connections, whereas a minority were highly connected. For the live trade network, districts located across the cattle corridor were often classified as central, either as districts with access to the global network and at highest risk of disease infection, or as districts at higher risk of pathogen exposure and onward disease transmission. Similar observations were made for the slaughter network, in which cattle slaughter tends to occur near major consumption urban centres. This structure may be an indication of asymmetric domestic supply–demand ratios for live animals, meat and meat products and suggests the existence of trading and slaughtering hubs. Therefore, systematic targeting of the most connected and influential districts would lead to the rapid fragmentation of the live trade network for cattle across the country, reducing the likelihood of disease spread^59,60^. Our percolation analysis supports the use of centrality-targeting to reduce the size of the largest connected component and can inform district prioritisation for animal health resource allocation (e.g., personnel, vaccines, etc.). Centrality-targeting is one of the spatial strategies used for strategic implementation of disease control interventions and has shown to be superior to risk-targeting when seeking to reduce epidemic contagion, especially in resource-constrained areas affected by epidemics caused by highly transmissible pathogens^61,62^, as is the case of FMD and other TADs affecting livestock^63^. For instance, spatial vaccination strategies informed by centrality-based prioritisation have shown to be effective at halting epidemic spread by reducing the size of the largest connected component, total number of individuals infected and the maximum number of simultaneous infections during an outbreak^64^.

Several large-scale disease control interventions could be implemented at the district level. An approach balancing the use of high-cost measures, such as pre-emptive vaccination or laboratory-based surveillance, could be used alongside resource-friendly methods for a multifaceted and cost-effective disease control strategy in areas where a lack of funds may be an issue. Strengthening community health education, reinforcing health checks, increasing biosecurity and hygienic practices, and implementing vehicle disinfection could be explored as strategies that can contribute to curtailing the probability of direct and indirect disease transmission when applied at mass scale, based on a trade-risk district prioritisation system. As the backbone structure of cattle trading in Uganda, livestock markets and nearby areas should be prioritised due to their importance as transmission hotspots for TADs and infectious diseases of public health significance^14,55,56^. While the acceptability, feasibility, effectiveness of such measures in empirical settings require further investigation, biosecurity measures and other health-related interventions have high uptake potential among farmers and traders^65,66^. These measures may also ease the unintended socio-economic effects that lengthy movement bans, and market closures have on the local communities, as reported for post-outbreak FMD measures implemented in similar environments^67^. In addition, slaughter hubs could be targeted for genomic surveillance and enhanced disease inspection and biosecurity. This would serve the dual function of hindering further disease transmission and early identification of infectious threats, enhancing epidemic preparedness and response. Evidence of the use of slaughterhouse as sources of data for animal health intelligence exists for FMD in Vietnam^31^, Shiga Toxigenic *Escherichia coli* in Nigeria^68^, Crimean Congo Haemorrhagic Fever (CCHF) in Kenya^69^, and liver fluke in Scotland^70^. Smart disease monitoring could be integrated to other data sources to better quantify disease risk across the country^71^. For instance, bridging the genomic gap for TADs would contribute to understand the evolutionary dynamics and diffusion patterns of relevant pathogens, and to better represent the genetic variability required for high-quality and cost-effective vaccine production.

The extent to which the cattle traffic data presented here represents the overall system or the real trading volumes, including the more informal beef value chain is uncertain. It is reasonable to believe that official AMPs are biased towards between-market livestock movements using major roads, from and to large ranches/farms, or slaughter movements destined to larger abattoirs where regulations are more likely to be enforced and larger volumes of cattle are traded. While the continuous district decentralisation may have contributed to low data availability in newly created districts^72^, difficulties in the collection of mobility data in some districts also arise from remoteness, lack of animal health personnel, or illegal trading and long-term grazing practices, which are difficult to record. For instance, the Karamoja region supports one of the largest nomadic pastoralist communities in Uganda, yet a very low proportion of AMPs were digitalised in this sub-region, despite having one of the most vibrant marketing systems with several known active trading routes operating across the country^40^. The Karamojong trek vast areas encompassing multiple districts in search of water and pastures for their livestock, often engaging in trading along the way without formal registry. Furthermore, international cattle trade and cross-border grazing movements frequently occur in borderline districts. However, daily husbandry-related movements, international routes, or within-district (between sub-counties) movements were not explored in this analysis. The last, partially due to issues in reconstructing the network at lower levels of node granularity. A combination of these aspects could have been linked to scarce cattle movement data in some districts located in the northwest, southeast and northeast. The comprehensiveness of official livestock movement data has been examined in prior reports using official data for static and dynamic network analysis and modelling in similar settings^73,74^.

Due to the intrinsic limitations of the current paper-based data collection system and the uncertainties of how well the data represent the market chains, our results should be interpreted with caution. A precise representation of livestock networks, based on a sensitive, specific, and accurate movement monitoring system should be developed and enforced to obtain high-quality, granular data for network analysis and dynamic disease modelling. An electronic data capture system could be adopted for real-time, centralised, robust digitised data recording, with numerous socio-economic and animal health benefits anticipated for more reliable animal movement information. Currently, such systems are being piloted in Uganda, mostly in connection to animal health and welfare, food safety and value chains, towards strengthening meat export endeavours^75,76^, but these are far from being implemented widely across the country.

The network analysis presented here offers insight into the structure of cattle mobility in Uganda. By analysing the temporal patterns of country-wide cattle shipments for the purpose of trade and slaughter, we have extended the scope of the analyses by Mugezi et al. and Hasahya et al.^34,77^. These analyses focused on qualitatively assessing the risk of FMD during the dry season through pastoral and trade-related cattle mobility along the Ugandan-Tanzanian border and on studying the patterns of live trade livestock movements (cattle, small ruminants, and pigs) in the cattle corridor of Uganda. We have highlighted the importance of seasonality on cattle trade and discussed the possible disease risks derived from local and long-haul live and slaughter cattle traffic across the country. However, a longitudinal, long-standing analysis of cattle shipments in Uganda is still required to confirm if the seasonal patterns identified here are consistent across the years. We have also informed the geographical locations of greatest interest for smart allocation of resources towards a feasible, timely, sustainable, and cost-effective planning for optimised disease preparedness and response. Tailored disease control interventions, adjusted to the agricultural reality of trading communities, should be prioritised in local hubs predominantly within the agro-pastoralist cattle corridor and the small-holder communities in peripheral districts along the west and southeast. This may contribute to reducing the burden of selected TADs with positive implications for the local cattle-raising communities and the wider agricultural socio-economic system in the country.

Further analysis should focus on multilayer networks of all livestock movements, including local grazing and transhumance mobility, dynamic modelling of disease and the effect of targeted disease control interventions considering the variations arising from the local livestock keeping systems, possible transmission routes, disease shedding cycles, livestock contact rates, and the epidemic calendar of selected TADs. Empirical evidence of the acceptability, feasibility, and effectiveness of interventions aimed at disconnecting the cattle networks should also be the aim of future investigations. This information, coupled with the analysis of grazing-oriented livestock movements, is required for a holistic understanding of cattle traffic and its potential effect on disease risk across the country.

### Supplementary Information

Supplementary Figures.

Supplementary Tables.

## Data Availability

The data that support the findings of this study are available from the Ministry of Agriculture, Animal Industry and Fisheries (MAAIF) in Uganda. Restrictions apply to the availability of these data, which were used under license for this study.
