# Supplementary material for: Identifying target areas for risk-based surveillance and control of transboundary animal diseases: a seasonal analysis of slaughter and live-trade cattle movements in Uganda
Source: Sci Rep. 2023 Oct 30;13:18619. doi: 10.1038/s41598-023-44518-4 (PMC10616094; doi:10.1038/s41598-023-44518-4)

Supplementary Information
Figures

Identifying target areas for risk-based surveillance and control of Transboundary Animal Diseases: A seasonal analysis of slaughter and live-trade cattle movements in Uganda

Lina González-Gordon[1,2*], Thibaud Porphyre[3], Adrian Muwonge[1,4], Noelina Nantima[5], Rose Ademun[5], Sylvester Ochwo[6], Norbert Frank Mwiine[7], Lisa Boden[2], Dennis Muhanguzi[7], Barend Mark de C Bronsvoort[1]

[1]The Epidemiology, Economics and Risk Assessment (EERA) Group, The Roslin Institute at The Royal (Dick) School of Veterinary Studies, University of Edinburgh, Easter Bush, Midlothian, EH25 9RG, United Kingdom.
[2] Global Academy of Agriculture and Food Systems, Royal (Dick) School of Veterinary Studies and The Roslin Institute, University of Edinburgh, Easter Bush, Midlothian, EH25 9RG, United Kingdom.
[3]Laboratoire de Biométrie et Biologie Évolutive, UMR 5558, Universite Claude Bernard Lyon 1, CNRS, VetAgro Sup, Marcy l'Étoile, France.
[4]The Digital One Health Laboratory, The Roslin Institute at The Royal (Dick) School of Veterinary Studies, University of Edinburgh, Easter Bush, Midlothian, EH25 9RG, United Kingdom.
[5]Department of Animal Health, Ministry of Agriculture Animal Industry & Fisheries, Entebbe, Uganda.
[6]Center for Animal Health and Food Safety, College of Veterinary Medicine, University of Minnesota, Saint Paul, MN 55108, United States.
[7]Department of BioMolecular Resources and BioLaboratory Sciences (BBS), College of Veterinary Medicine, Animal Resources and Biosecurity, Makerere University, Kampala, Uganda.

Figure S1. Active districts for the live trade and slaughter cattle networks. Origin districts are highlighted in orange, whereas yellow is used to depict destination districts for each network.

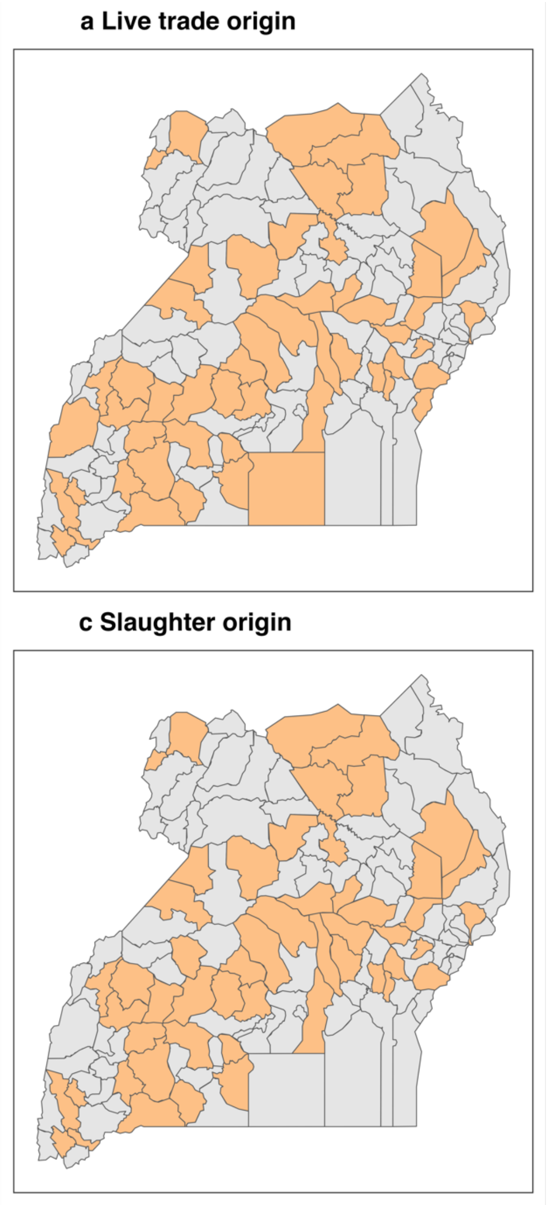

Figure S2. Network visualisation of node and edge fidelity across the seasons. Districts of origin are highlighted in each of seasonal networks: (1) Live trade and (2) Slaughter.

(1) Live trade

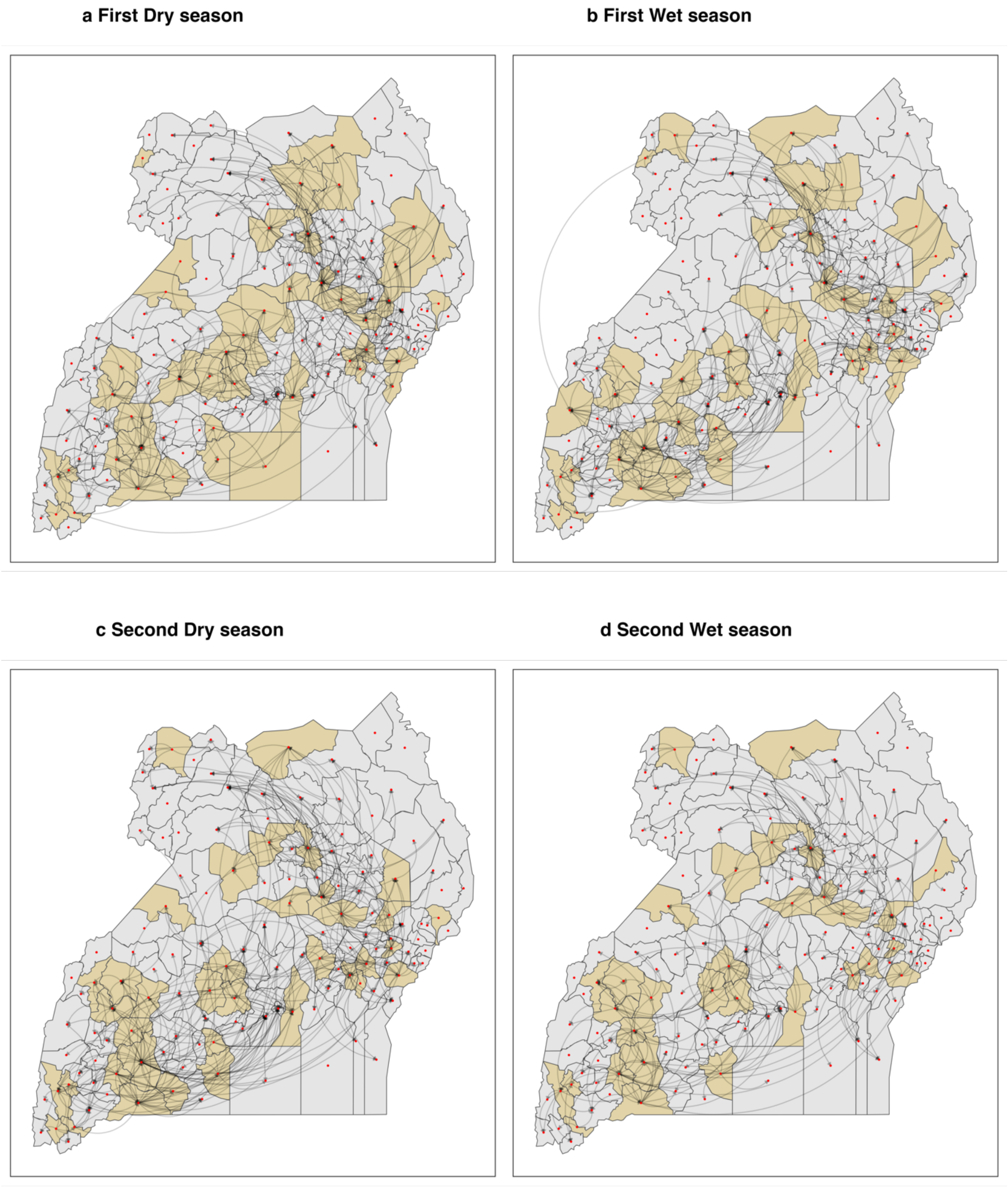

(2) Slaughter

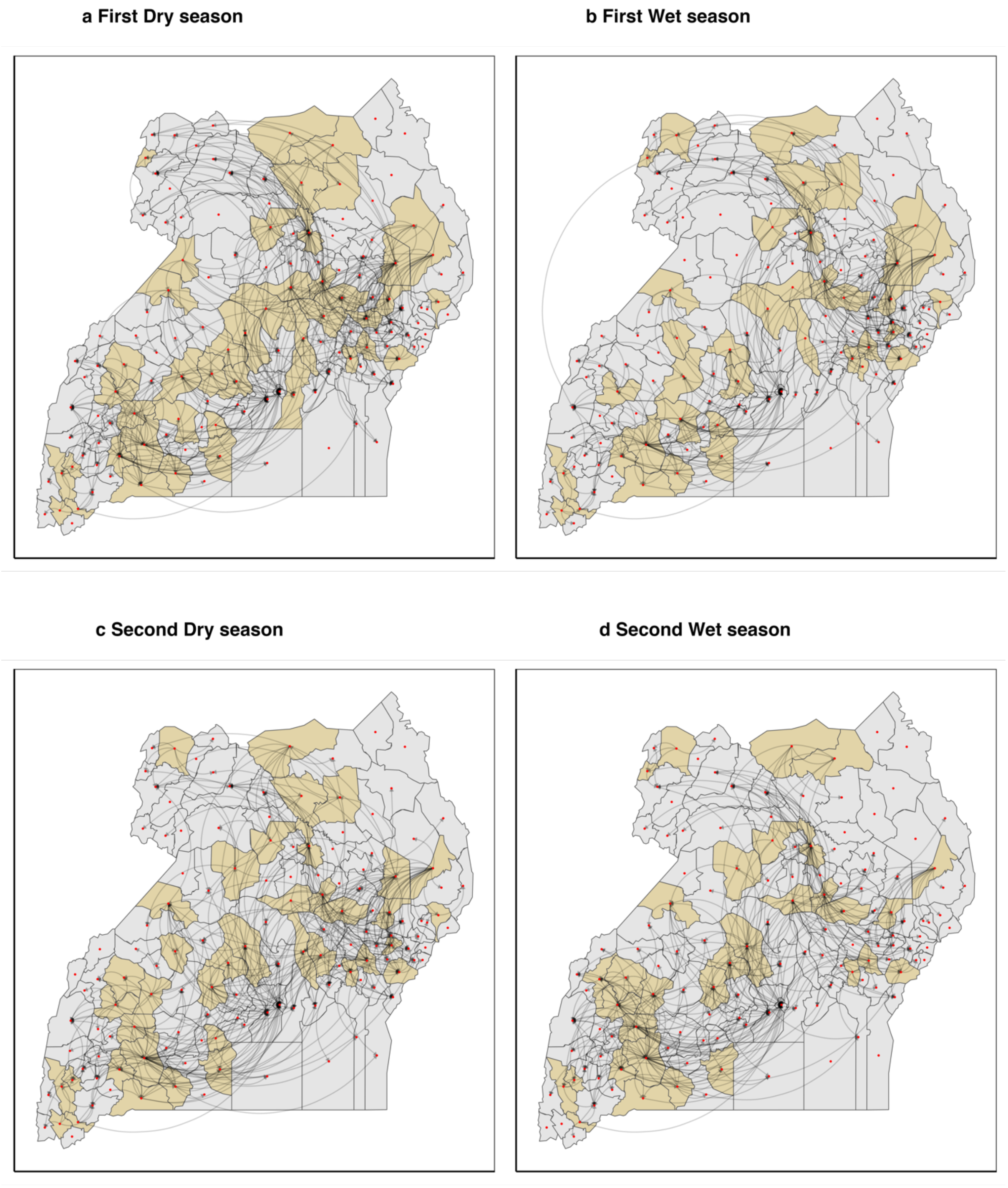
a First Dry season
b First Wet season
c Second Dry season
d Second Wet season

Figure S3. Slaughter network communities and its spatial distribution across the seasons. Districts in grey were not active in the network for the period. Each community is depicted by a unique colour.

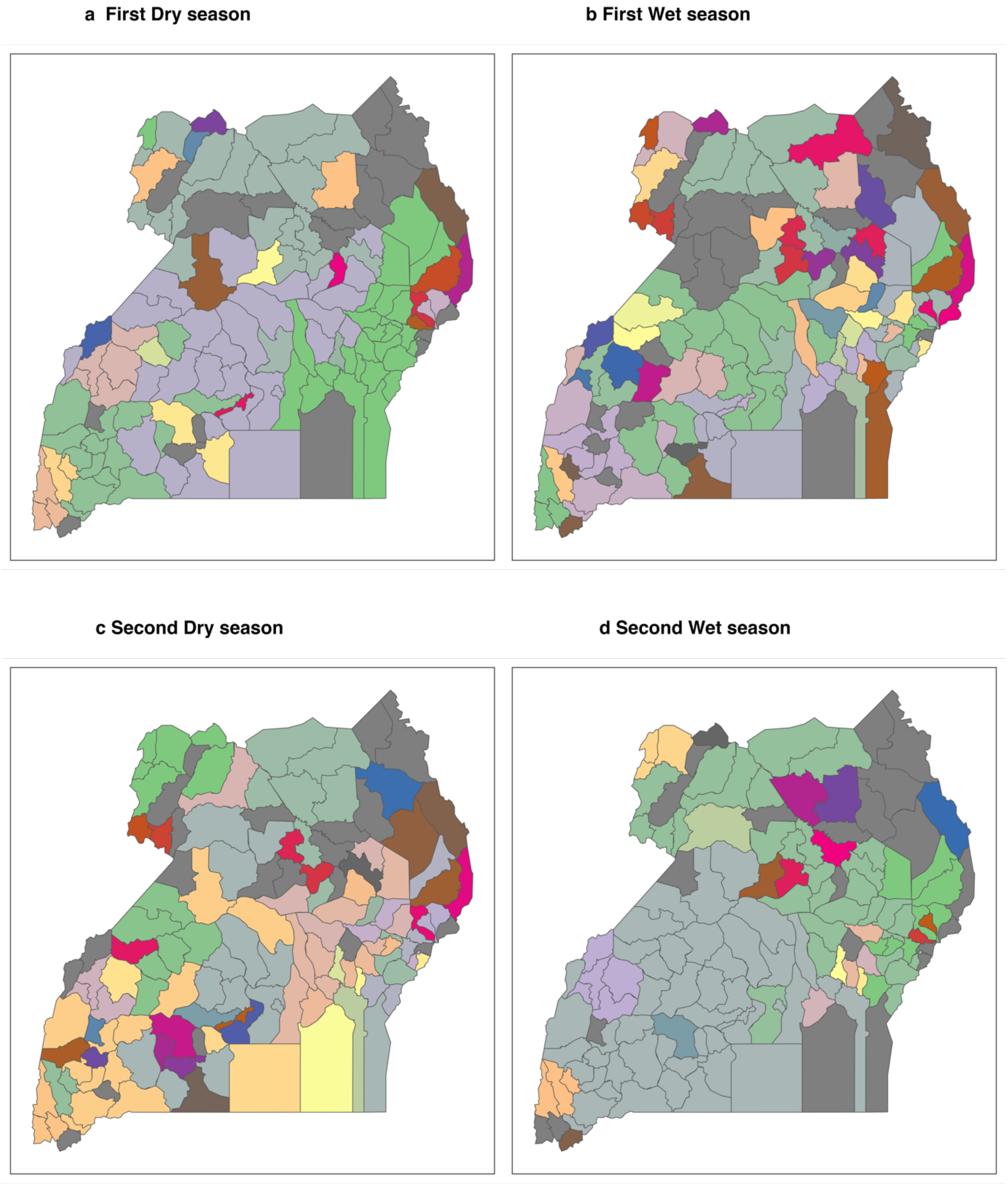

Figure S4. Analysis of the strength and degree centrality across seasons: Live trade (A - B) and slaughter (C - D) networks.

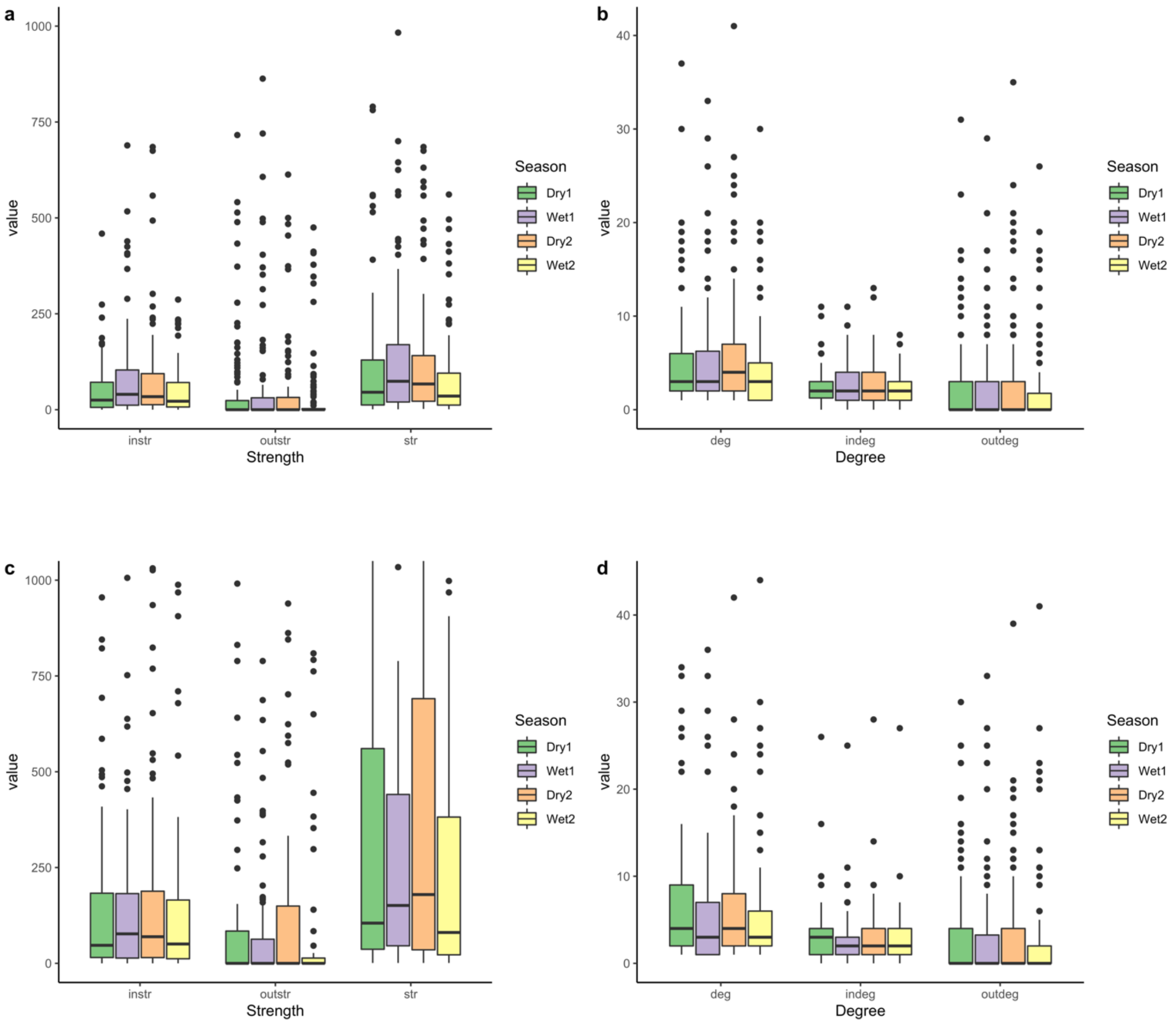

Figure S5. Geographical distribution of top-ranked central districts per season – Live trade network. For each measure, top 5% districts coloured in red, 5-10% in blue and all the other districts in green.

(a) In-degree

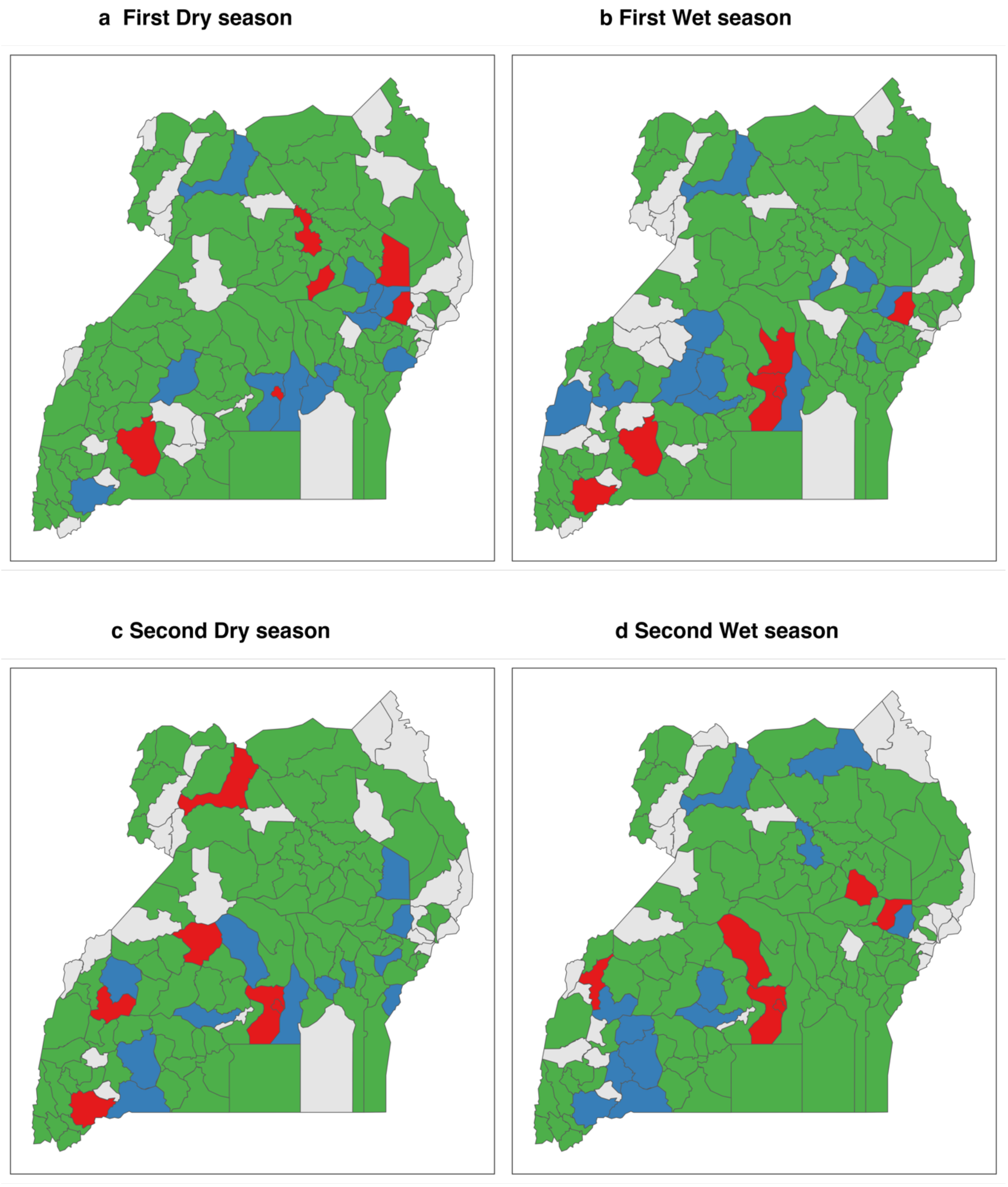

(b) Out-degree

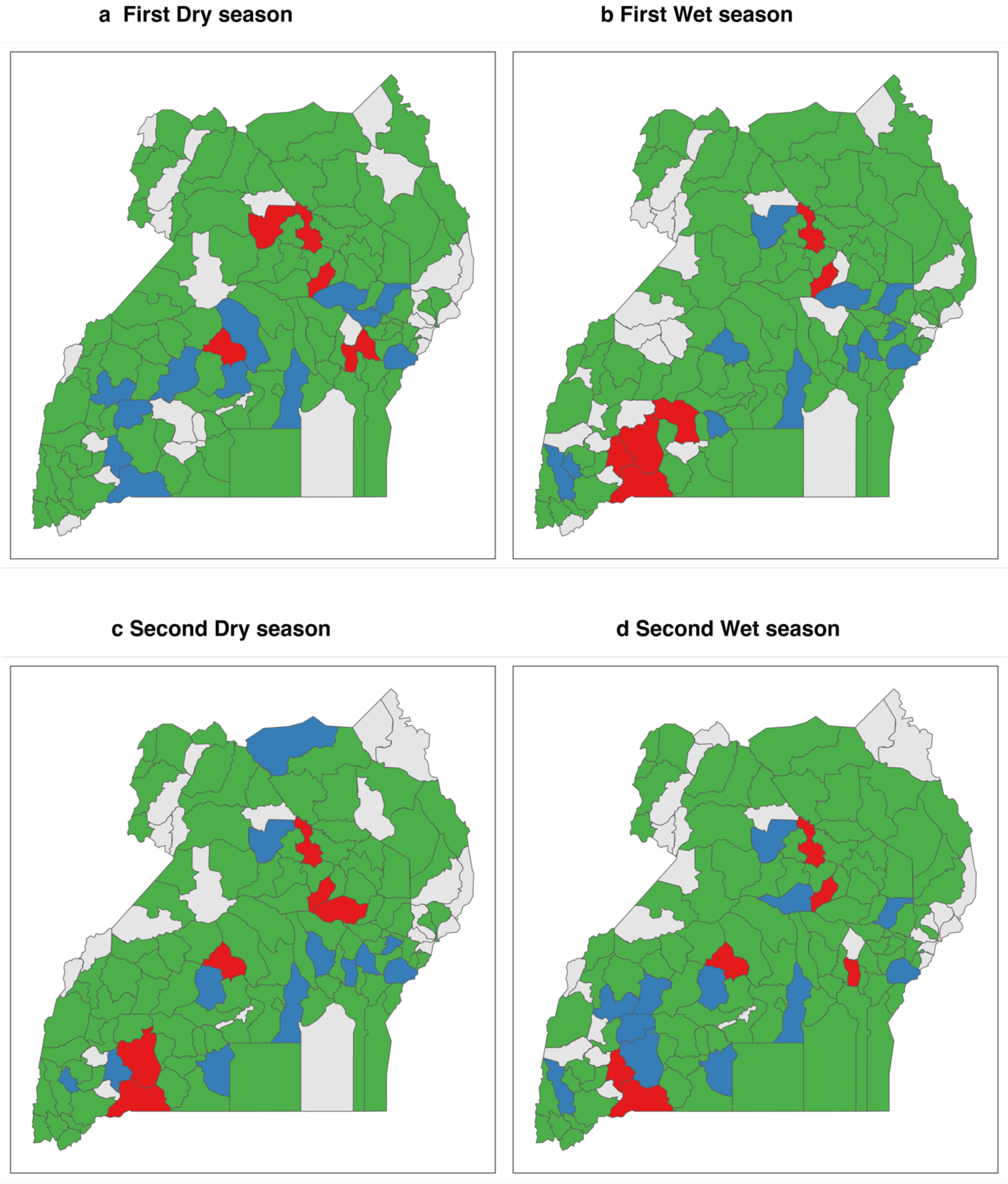
a First Dry season
b First Wet season
c Second Dry season
d Second Wet season

(c) In-strength

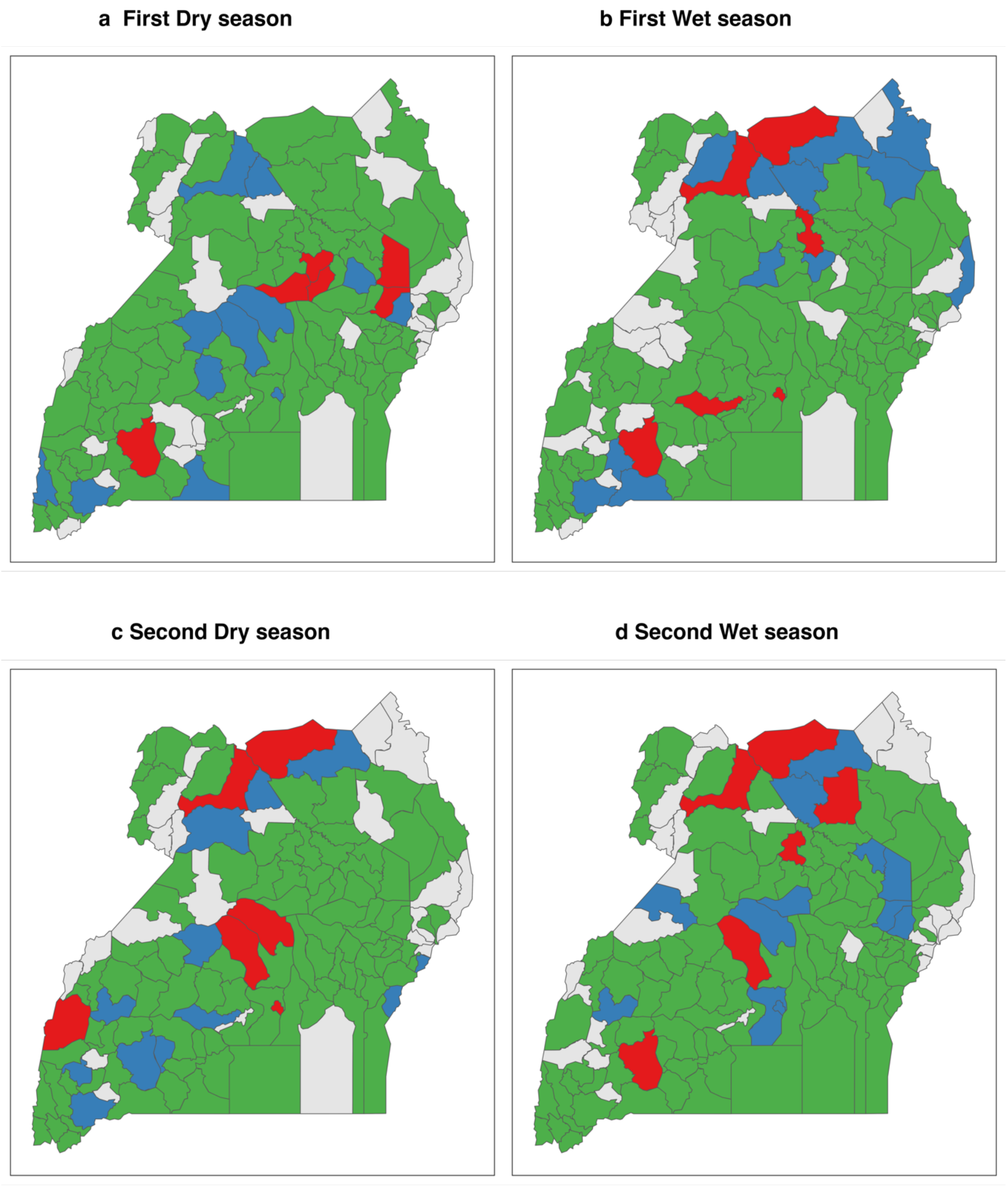

(d) Out-strength

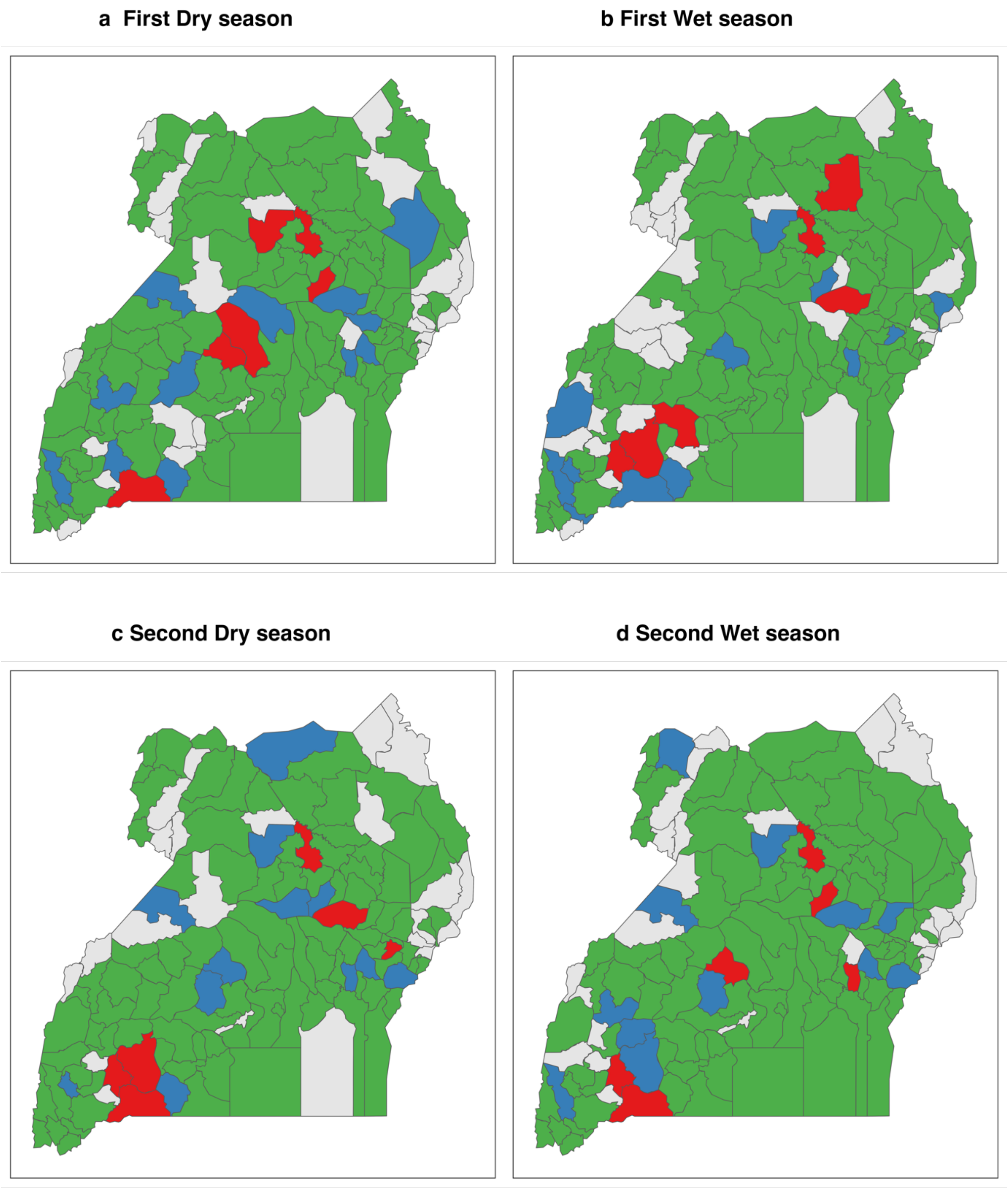

(e) Betweenness

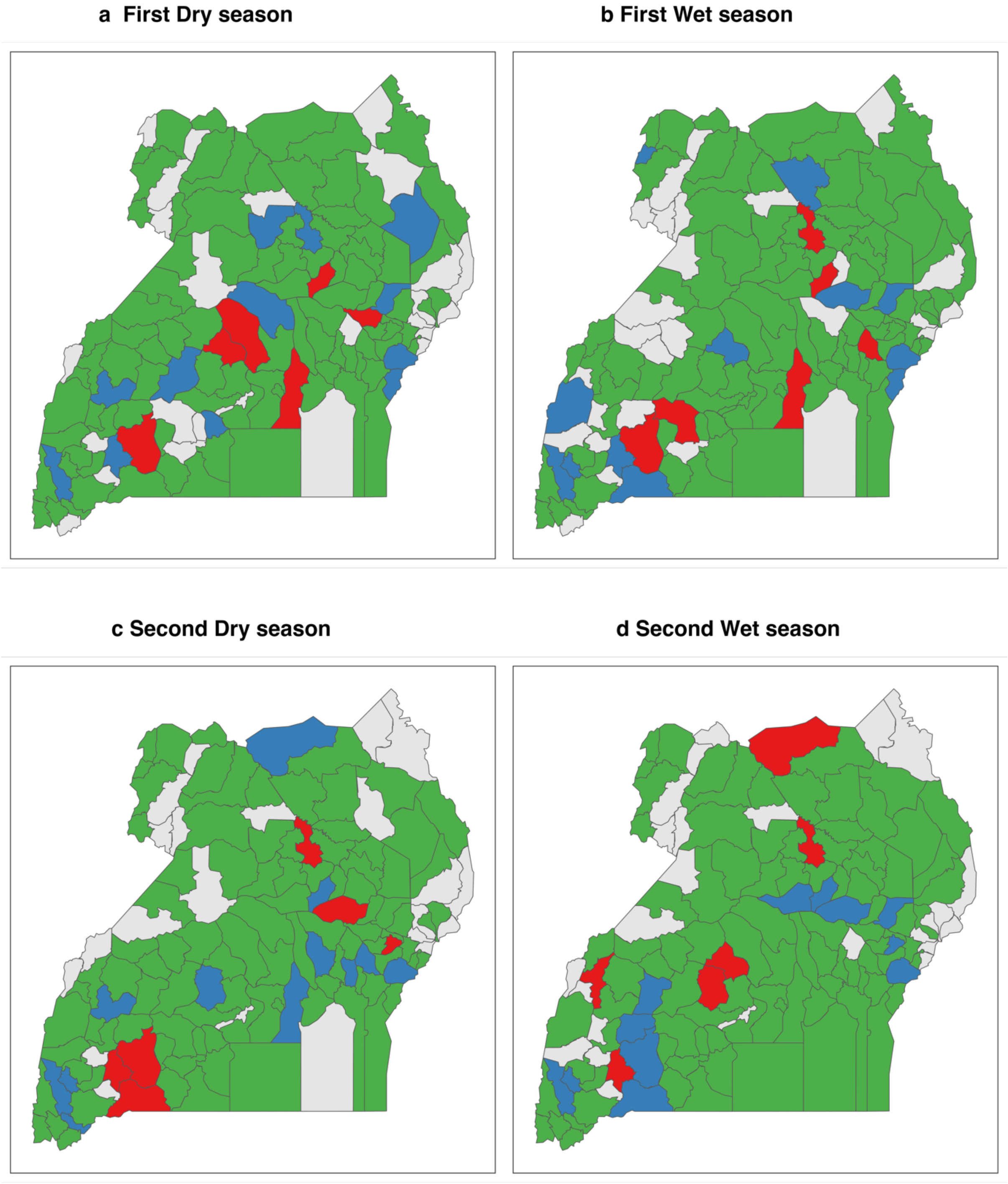

(f) Page-rank

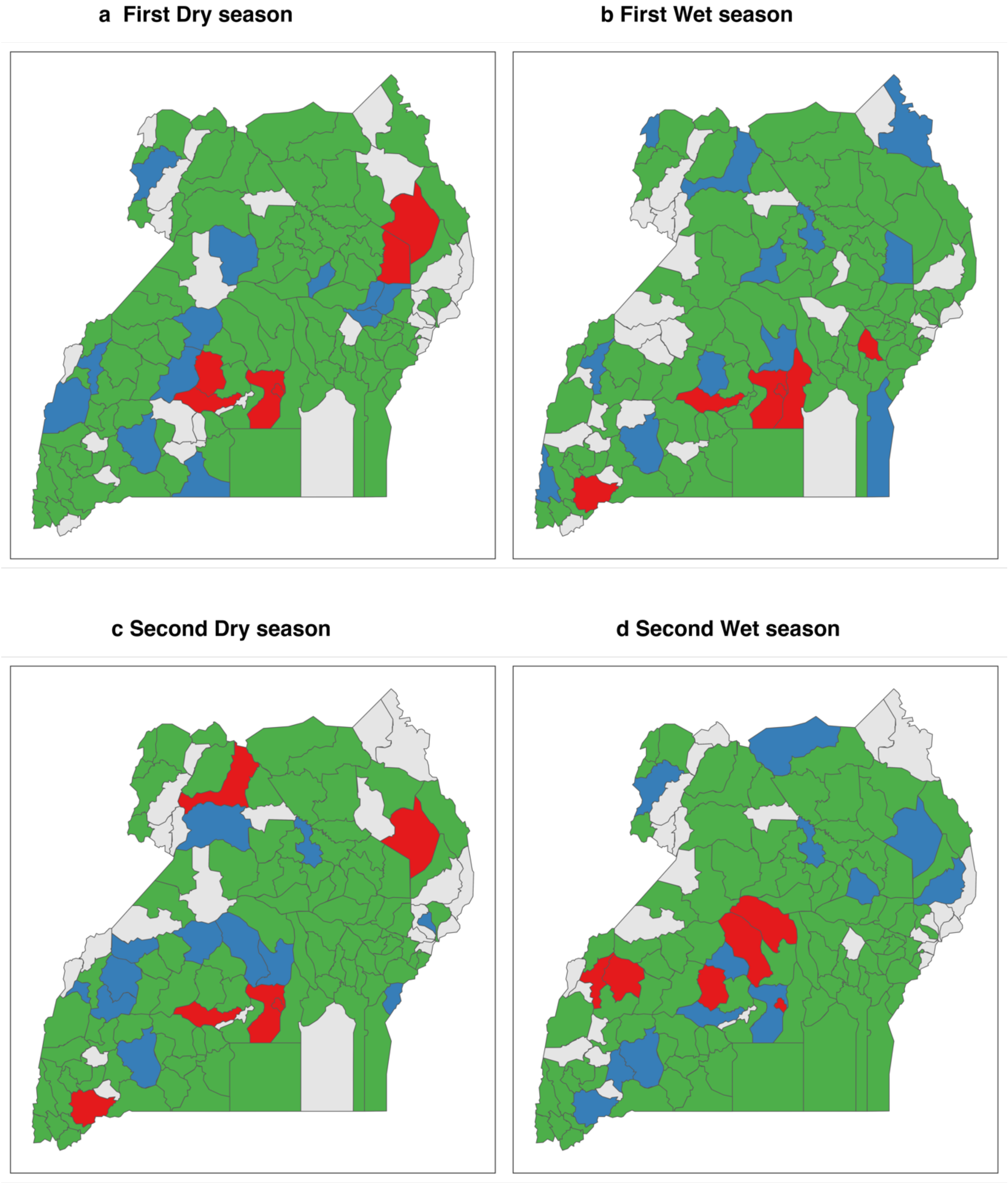
a First Dry season
b First Wet season
c Second Dry season
d Second Wet season

Figure S6. Geographical distribution of top-ranked central districts per season – Slaughter network. For each measure, top 5% districts coloured in red, 5-10% in blue and all the other districts in green.

(a) In-degree

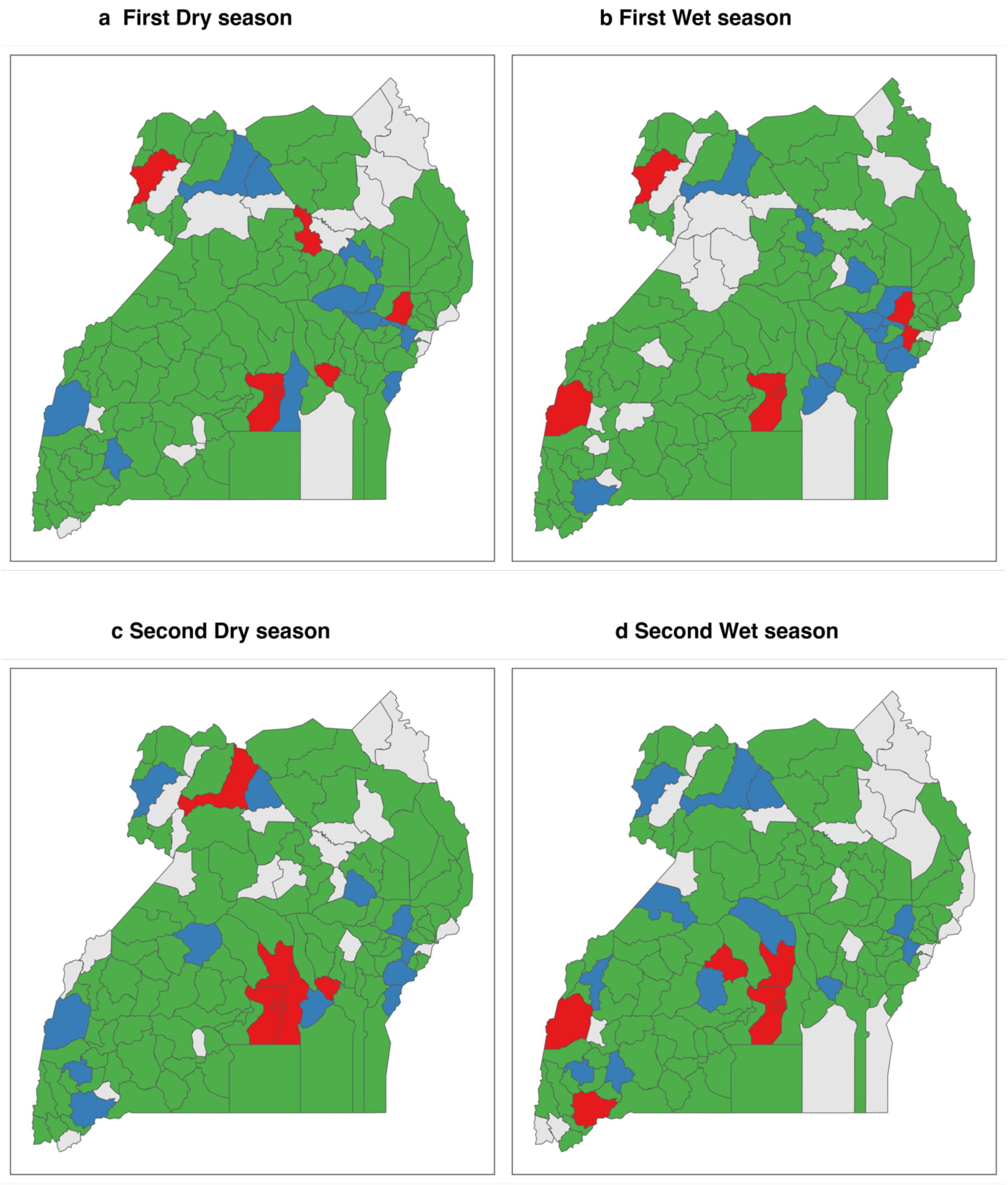

(b) Out-degree

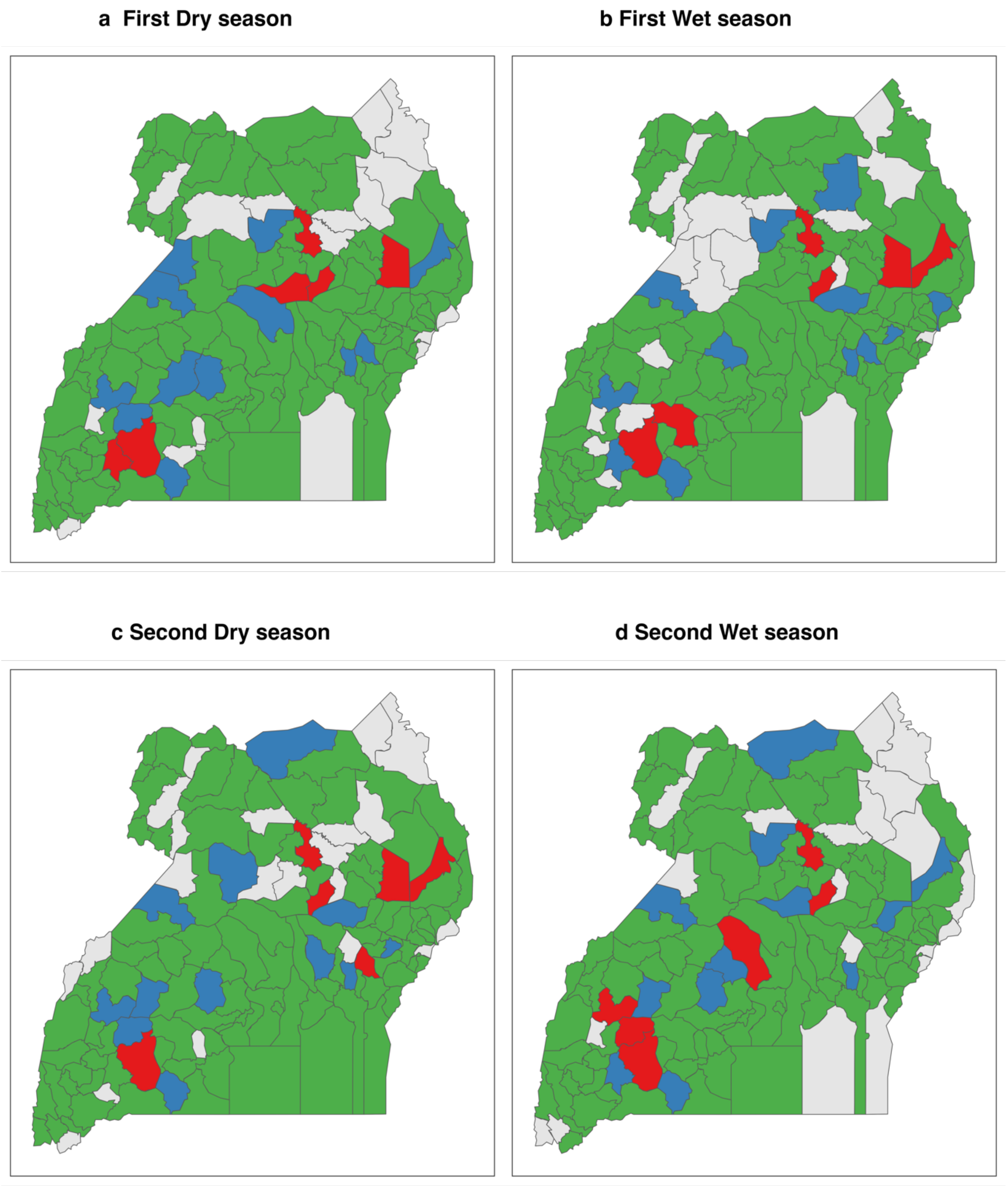

a First Dry season
b First Wet season
c Second Dry season
d Second Wet season

(c) In-strength

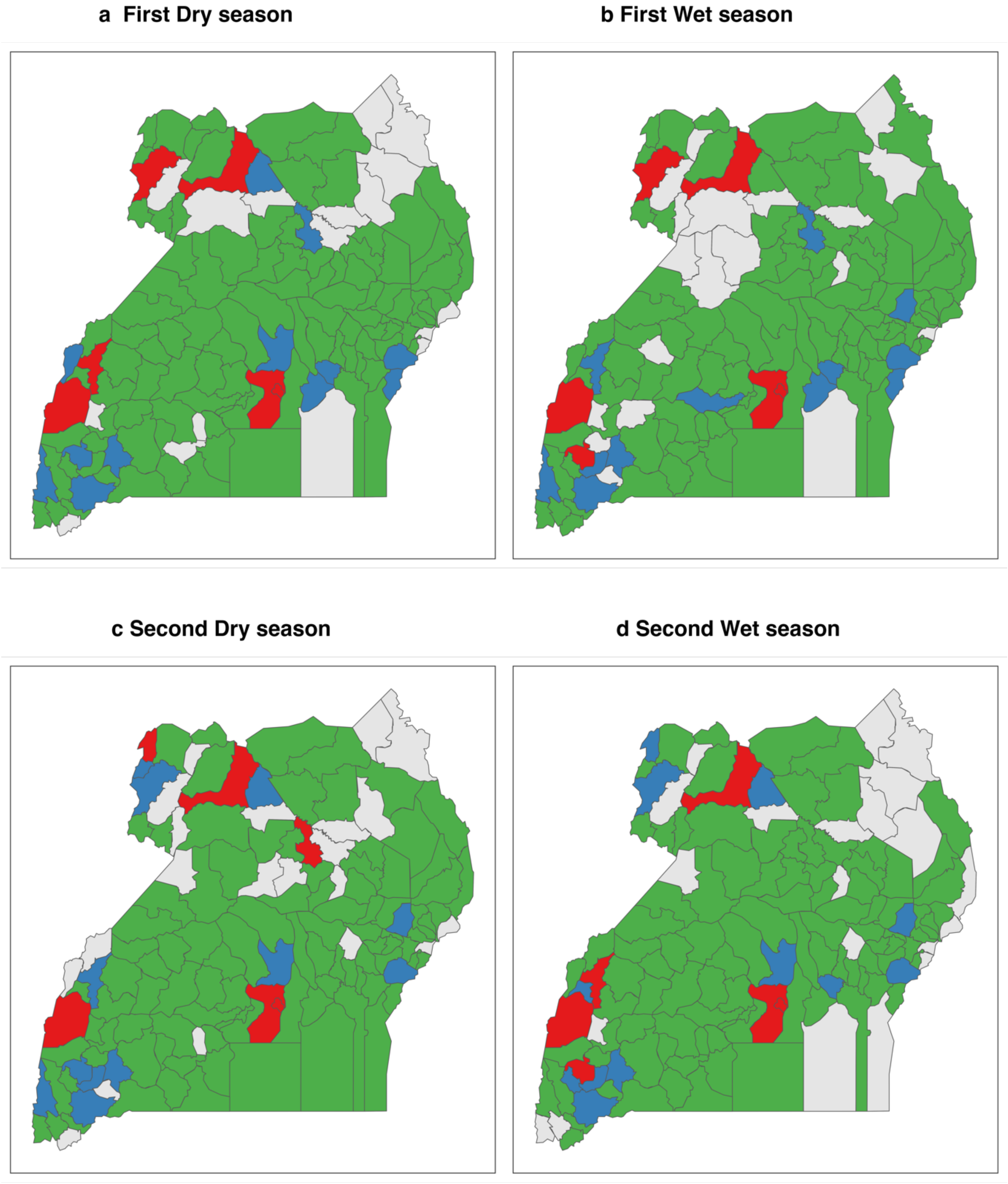

(d) Out-strength

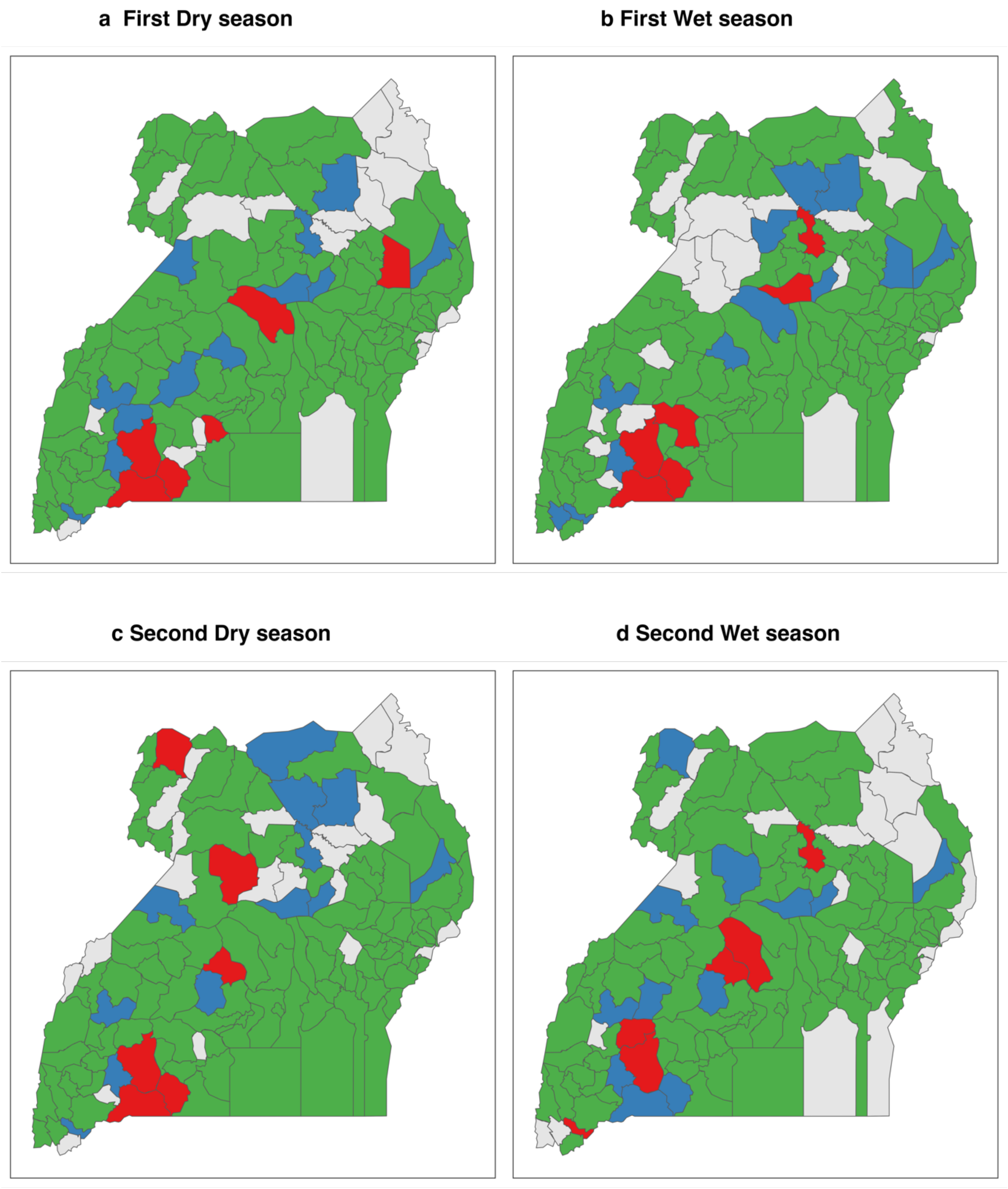

Figure S7. Plot of centrality-based percolation per season – Live trade. Performance measured through the proportional size of the giant weakly connected component (GWCC).

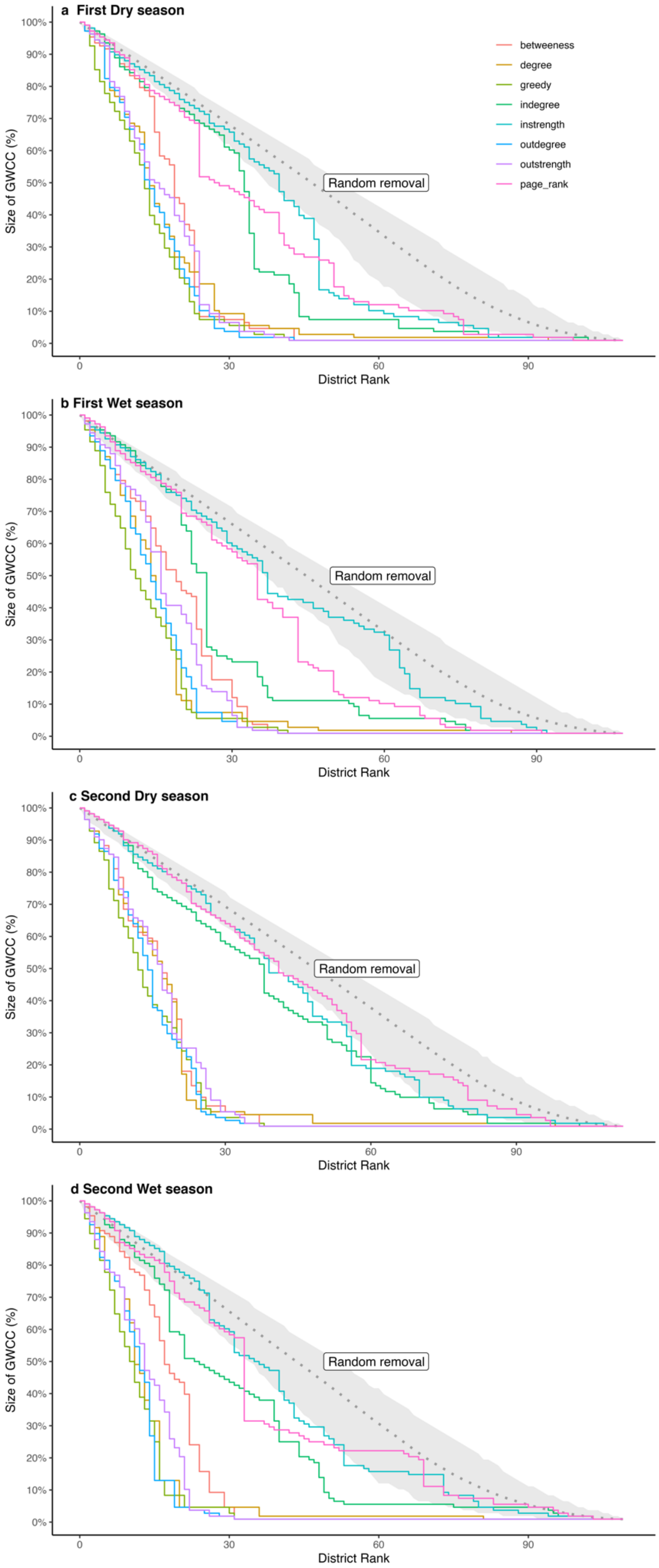

Supplement: Supplementary file 1 — Supplementary Figures. [file 41598_2023_44518_MOESM1_ESM.pdf]
