# Supplementary material for: Identifying target areas for risk-based surveillance and control of transboundary animal diseases: a seasonal analysis of slaughter and live-trade cattle movements in Uganda
Source: Sci Rep. 2023 Oct 30;13:18619. doi: 10.1038/s41598-023-44518-4 (PMC10616094; doi:10.1038/s41598-023-44518-4)
Supplement: Supplementary file 2 — Supplementary Tables. [file 41598_2023_44518_MOESM2_ESM.pdf]

Supplementary Information
Tables

Identifying target areas for risk-based surveillance and control of Transboundary Animal Diseases: A seasonal analysis of slaughter and live-trade cattle movements in Uganda

Lina González-Gordon[1,2*], Thibaud Porphyre[3], Adrian Muwonge[1,4], Noelina Nantima[5], Rose Ademun[5], Sylvester Ochwo[6], Norbert Frank Mwiine[7], Lisa Boden[2], Dennis Muhanguzi[7], Barend Mark de C Bronsvoort[1]

[1]The Epidemiology, Economics and Risk Assessment (EERA) Group, The Roslin Institute at The Royal (Dick) School of Veterinary Studies, University of Edinburgh, Easter Bush, Midlothian, EH25 9RG, United Kingdom.
[2] Global Academy of Agriculture and Food Systems, Royal (Dick) School of Veterinary Studies and The Roslin Institute, University of Edinburgh, Easter Bush, Midlothian, EH25 9RG, United Kingdom.
[3] Laboratoire de Biométrie et Biologie Évolutive, UMR 5558, Universite Claude Bernard Lyon 1, CNRS, VetAgro Sup, Marcy l'Étoile, France.
[4]The Digital One Health Laboratory, The Roslin Institute at The Royal (Dick) School of Veterinary Studies, University of Edinburgh, Easter Bush, Midlothian, EH25 9RG, United Kingdom.
[5]Department of Animal Health, Ministry of Agriculture Animal Industry & Fisheries, Entebbe, Uganda.
[6]Center for Animal Health and Food Safety, College of Veterinary Medicine, University of Minnesota, Saint Paul, MN 55108, United States.
[7]Department of BioMolecular Resources and BioLaboratory Sciences (BBS), College of Veterinary Medicine, Animal Resources and Biosecurity, Makerere University, Kampala, Uganda.

Table S1. Network topology and cohesiveness parameter definition and interpretation**.**

	Parameter	Definition
**Network topology (or architecture)**	**Node**	Unit of analysis in a network – Uganda's districts
	**Arcs**	Directed district-to-district links representing animal movements.
	**Edges**	Non-directed district-to-district links representing animal movements.
	**Diameter**	The longest 'shortest path length' between any pair of districts in the network.
	**Density**	Proportion of links (edges) between districts that are present out of the maximum possible for a fully connected all-country network.
	**Reciprocity**	Proportion of mutual connections between districts.
	**Degree assortativity**	Quantifies the tendency of districts to connect with districts of similar degree.
**Network cohesiveness**	**Average path length**	Shortest path between any two districts across the network over all pairs of districts within the network.
	**Transitivity (or Clustering coefficient)**	Probability that neighbouring districts are connected forming triads, quantifies the abundance of clusters within the network.
	**Components**	Groups formed by paths connecting nodes across the network – Named 'Giant Weakly Connected Components (GWCC)' if non-directed and 'Giant Strongly Connected Components' when districts can be reached via a directed path.
	**Community**	Identification of densely connected districts forming sub-groups. Also called clusters or modules.
	**Modularity**	Measure indicating how separated are communities from one another; high modularity suggests dense within-community links with sparse between-community links.

Table S2. Between-district cattle movements associated with live trade and slaughter in Uganda. 2019.

	Live trade		Slaughter	
	**Movement records**	**Number of animals**	**Movement records**	**Number of animals**
**Transport (n, %)**				
Land	5,106 (99.84)	28,914 (99.96)	16,863 (99.72)	205,102 (99.90)
Water	8 (0.16)	13 (0.04)	47 (0.28)	200 (0.1)
**Means of transport (n, %)**				
Truck	3,128 (61.17)	24,718 (85.45)	14,476 (85.61)	194,482 (94.73)
Car, Motorcycle & Bicycles	1,978 (38.67)	4,196 (14.51)	2,387 (14.12)	10,620 (5.18)
Boat	8 (0.16)	13 (0.04)	47 (0.28)	200 (0.10)
**Purpose (n, %)**				
Breeding	5,113 (99.98)	28,904 (99.92)	-	-
Other (e.g., ploughing)	1 (0.02)	23 (0.08)	-	-
**Sex* (n, %)**				
Male	2,404 (47.67)	10,052 (34.96)	9,849 (58.26)	117,066 (57.02)
Female	2,639 (52.33)	18,700 (65.04)	7,057 (41.74)	88,229 (42.98)
**Age* (n, %)**				
Adult	4,132 (81.94)	24,086 (83.77)	16,488 (97.53)	199,290 (97.07)
Young	911 (18.06)	4,666 (16.23)	418 (2.48)	6,005 (2.93)

* Missing information for a proportion of movement records.

Table S3. Comparison of network features between the yearly and seasonal country-wide cattle movement networks, live trade, 2019.

	Live-trade network				
	**Annual**	**Dry season 1 (Dec–Feb)**	**Wet season 1 (Mar–May)**	**Dry season 2 (Jun–Aug)**	**Wet season 2 (Sep–Nov)**
**Movement records**	5,114	1,169	1,425	1,434	1,086
**Cattle numbers**	28,927	5,724	9,106	8,818	5,279
**Topology**					
Node	125	110	108	113	110
Arcs	687	305	295	335	239
Diameter	8	12	13	7	9
Density	0.04	0.02	0.02	0.02	0.01
Reciprocity	0.17	0.09	0.08	0.05	0.10
Degree assortativity	-0.15	-0.10	-0.06	-0.12	-0.12
**Cohesiveness**					
Average path length	2.69	3.78	3.52	2.86	3.38
Transitivity	0.27	0.20	0.21	0.18	0.11
WCC	1	2	1	2	2
GWCC	125	108	108	111	108
SCC	78	81	86	95	96
GSCC	48	28	22	9	8
Communities	11	17	20	18	18
Modularity	0.48	0.53	0.54	0.52	0.57

Table S4. Comparison of network features between the yearly and seasonal country-wide cattle movement networks, slaughter, 2019.

	Slaughter network				
	**Annual**	**Dry season 1 (Dec–Feb)**	**Wet season 1 (Mar–May)**	**Dry season 2 (Jun–Aug)**	**Wet season 2 (Sep–Nov)**
**Movement records**	16,910	3,718	4,111	4,924	4,157
**Cattle numbers**	205,302	44,433	53,569	54,175	53,125
**Topology**					
Node	130	118	116	114	114
Arcs	716	384	319	364	325
Diameter	2	2	2	2	2
Density	0.04	0.02	0.02	0.02	0.02
Reciprocity	0.12	0.06	0.06	0.04	0.05
Degree assortativity	-0.16	-0.09	-0.19	-0.13	-0.19
**Cohesiveness**					
Average path length	2.91	5.14	2.64	3.18	2.83
Transitivity	0.27	0.20	0.20	0.19	0.18
WCC	1	1	1	1	1
GWCC	130	118	116	114	114
SCC	91	95	103	94	101
GSCC	39	24	9	17	13
Communities	22	24	60	45	22
Modularity	0.27	0.42	0.32	0.39	0.22

Table S5. Small-worldness index computation.

	Live trade					Slaughter				
	Annual	Dry 1 (Dec – Feb)	Wet 1 (Mar – May)	Dry 2 (Jun –Aug)	Wet 2 (Sep – Nov)	Annual	Dry 1 (Dec – Feb)	Wet 1 (Mar – May)	Dry 2 (Jun- Aug)	Wet 2 (Sep – Nov)
**Transitivity**	0.27	0.20	0.21	0.18	0.11	0.27	0.20	0.20	0.19	0.18
**Transitivity random**	0.04	0.021	0.02	0.021	0.01	0.04	0.02	0.02	0.02	0.02
**Average path length**	2.69	3.78	3.52	2.86	3.38	2.91	5.14	2.64	3.18	2.83
**Average path length random**	2.99	4.54	4.58	4.31	5.81	3.01	4.05	4.62	4.08	4.46
**Index**	6.88	9.52	10.91	10.48	9.96	6.66	5.74	15.09	9.00	11.92

Table S6. Degree and strength centralities for live trade and slaughter cattle network.

	Live trade						Slaughter					
**Season**	**Degree**	**In-degree**	**Out-degree**	**Strength**	**In-strength**	**Out-strength**	**Degree**	**In degree**	**Out degree**	**Strength**	**In strength**	**Out strength**
**Dry 1**												
**Me [Min-Max]**	3 [1-37]	2 [0-11]	0 [0-31]	45.5 [1-790]	25 [0-459]	0 [0-716]	4 [1-34]	3 [0-26]	0[0-30]	105 [1-20,830]	47 [0-20,830]	0 [0-4,776]
**Average**	5.54	2.77	2.77	104.07	52.04	52.04	6.50	3.25	3.25	753	376.55	376.55
**Wet 1**												
**Me [Min-Max]**	3 [1-33]	2 [0-11]	0 [0-29]	74 [1-3,143]	40 [0-689]	0 [0-2,626]	3 [1-36]	2 [0-25]	0 [0-33]	151 [1-24,450]	77 [0-24,450]	0 [0-11,352]
**Average**	5.46	2.73	2.73	168.6	84.31	84.31	5.5	2.75	2.75	923	461.80	461.80
**Dry 2**												
**Me [Min-Max]**	4 [1-41]	2 [0-13]	0 [0-35]	67 [2-3,087]	34 [0-685]	0 [0-2,863]	4 [1-42]	2 [0-28]	0 [0-39]	179.5 [1-24,798]	69.5 [0-24,798]	0 [0-12,530]
**Average**	5.92	2.96	2.96	156.1	78.04	78.04	6.38	3.19	3.19	950.44	475.2	475.2
**Wet 2**												
**Me [Min-Max]**	0 [1-30]	2 [0-8]	0 [0-26]	35 [1-1,610]	22 [0-287]	0 [0-1,572]	3 [1-44]	2 [0-27]	0 [0-41]	80.5 [1-23,545]	50.5 [0-23,545]	0 [0-13]
**Average**	4.35	2.17	2.17	95.25	47.99	47.99	5.70	2.85	2.85	932.02	466	466.01
**2019**												
**Me [Min-Max]**	7 [1-64]	5 [0-25]	0 [0-48]	217 [1-6,963]	137 [0-1,318]	0 [0-6,232]	8 [1-68]	5 [0-42]	0 [0-61]	528 [1-93,623]	217 [0-93,623]	0 [0-42,186]
**Average**	10.99	5.49	5.49	462.8	231.4	231.4	11.02	5.50	5.50	3,158	1,579.25	1,579.2

Table S7. Quantitative variables that describe each cluster – Live trade cattle network.

(a) First dry season – December to February

Cluster	No. districts	Variable	Mean in category	Overall mean	Interpretation
**1**	26	Local transitivity	1.43	9.77e-18	High local transitivity with low node centrality
		PageRank	-0.42	-6.72e-17	
		Out-strength	-0.40	-1.84e-17	
		Betweenness	-0.41	2.26e-17	
		Out-degree	-0.45	-7.95e-17	
**2**	33	Betweenness	-0.38	2.26e-17	Low node centrality
		PageRank	-0.58	-6.72e-17	
		In-strength	-0.63	-2.32e-17	
		Local transitivity	-0.89	9.77e-18	
		In-degree	-0.91	-1.20e-16	
**3**	29	In-degree	0.57	-1.20e-16	High in-degree, PageRank and in-strength with low betweenness, out-strength and out-degree
		PageRank	0.45	-6.72e-17	
		In-strength	0.42	-2.32e-17	
		Betweenness	-0.33	2.26e-17	
		Out-strength	-0.40	-1.84e-17	
		Out-degree	-0.46	-7.95e-17	
**4**	14	Out-degree	1.48	-7.95e-17	High out-degree, out-strength and betweenness
		Out-strength	1.31	-1.84e-17	
		Betweenness	1.30	2.26e-17	
**5**	5	PageRank	2.92	-6.72e-17	High PageRank, in-strength, in-degree and betweenness
		In-strength	2.61	-2.32e-17	
		In-degree	2.27	-1.20e-16	
		Betweenness	1.14	2.26e-17	
**6**	3	Out-strength	4.27	-1.84e-17	High out-strength, out-degree, in-degree and betweenness
		Out-degree	3.87	-7.95e-17	
		Betweenness	3.09	2.26e-17	
		In-degree	1.31	-1.20e-16	

(b) First wet season – March to May

Cluster	No. districts	Variable	Mean in category	Overall mean	Interpretation
**1**	54	Betweenness	-0.24	-2.28e-17	Low node centrality and low local transitivity
		PageRank	-0.50	-1.77e-16	
		In-strength	-0.54	5.85e-17	
		In-degree	-0.66	7.85e-17	
		Local transitivity	-0.65	-1.27e-17	
**2**	46	Local transitivity	0.85	-1.27e-17	High local transitivity, in-degree, in-strength, betweenness and PageRank with low out-strength and out-degree
		In-degree	0.62	7.85e-17	
		In-strength	0.48	5.85e-17	
		PageRank	0.42	-1.77e-16	
		Betweenness	-0.24	-2.28e-17	
		Out-strength	-0.26	6.20e-18	
		Out-degree	-0.41	4.09e-17	
**3**	9	Betweenness	3.04	-2.82e-17	High out-degree, out-strength, betweenness, PageRank, in-degree and in-strength
		Out-degree	2.58	4.09e-17	
		Out-strength	2.11	6.20e-18	
		PageRank	0.93	-1.77e-16	
		In-degree	0.87	7.85e-17	
		In-strength	0.85	5.85e-17	

(b) Second dry season – June to August

Cluster	No. districts	Variable	Mean in category	Overall mean	Interpretation
**1**	97	Out-strength	-0.18	3.52e-17	Low node centrality
		Out-degree	-0.20	3.27e-17	
		In-strength	-0.22	3.63e-17	
		PageRank	-0.23	-2.18e-16	
		Betweenness	-0.23	-2.34e-18	
		In-degree	-0.23	3.67e-17	
**2**	9	PageRank	2.34	-2.18e-16	High PageRank, in-strength and in-degree
		In-strength	2.33	3.63e-17	
		In-degree	2.28	3.67e-17	
**3**	8	Betweenness	3.10	-2.34e-18	High betweenness, out-degree and out-strength with low local transitivity
		Out-degree	2.91	3.27e-17	
		Out-strength	2.44	3.52e-17	
		Local transitivity	-0.67	1.14e-16	

(d) Second wet season – September to November

Cluster	No. districts	Variable	Mean in category	Overall mean	Interpretation
**1**	65	Out-strength	-0.21	-7.81e-18	Low node centrality and low local transitivity
		Out-degree	-0.22	-1.05e-17	
		Betweenness	-0.29	3.70e-17	
		PageRank	-0.37	6.20e-17	
		In-degree	-0.45	-8.64e-17	
		In-strength	-0.47	-2.81e-17	
		Local transitivity	-0.51	-4.69e-17	
**2**	16	Local transitivity	2.14	-4.69e-17	High Local transitivity
**3**	21	In-strength	1.56	-2.81e-17	High in-strength, in-degree, and PageRank
		In-degree	1.36	-8.64e-17	
		PageRank	1.21	6.20e-17	
**4**	9	Out-degree	2.65	-1.05e-17	High out-degree, out-strength and betweenness
		Betweenness	2.28	3.70e-17	
		Out-strength	2.26	-7.81e-18	
